# Safety, Tolerability, and Immunogenicity of RSVpreF Vaccine in Pregnant Individuals Living with HIV

**DOI:** 10.3390/vaccines13121218

**Published:** 2025-12-01

**Authors:** Landon Myer, Emily Wasserman, Saiqa Tabasum, Emma Shittu, Yanping Liu, Lisa Jose, Elizea Horne, Robert S. Moraba, Agatha Wilhase, Heather J. Zar, Nazreen Hussen, Mokgadi S. Mogashoa, Mookho Malahleha, Shabir A. Madhi, Uzma N. Sarwar, Hasra Snaggs, Rahsan Erdem, David Radley, Elena V. Kalinina, Barbara A. Pahud, Maria Maddalena Lino, Olympia Evdoxia Anastasiou, Kena A. Swanson, Annaliesa S. Anderson, Alejandra Gurtman, Iona Munjal

**Affiliations:** 1 Division of Epidemiology and Biostatistics, School of Public Health, University of Cape Town, Cape Town 7925, South Africa; 2 Pfizer Vaccines, Pfizer Inc., Pearl River, NY 10965, USA; 3 Pfizer Vaccines, Pfizer Ltd., Buckinghamshire, SL7 1YL, UK; 4 Pfizer Vaccines, Pfizer Inc., Collegeville, PA 19426, USA; 5 South African Medical Research Council, Vaccines and Infectious Diseases Analytics Research Unit, University of the Witwatersrand, Johannesburg 1864, South Africa; 6 Wits RHI, Faculty of Health Sciences, University of the Witwatersrand, Johannesburg 2001, South Africa; 7 Setshaba Research Centre, Tshwane City 0152, South Africa; 8 REIMED (Pty) Ltd., Johannesburg 1459, South Africa; 9 Department of Paediatrics and Child Health, Red Cross War Memorial Children’s Hospital, SA-MRC Unit on Child & Adolescent Health, University of Cape Town, Cape Town 8001, South Africa; 10 Worthwhile Clinical Trials, Benoni 1500, South Africa; 11 Botho Ke Bontle Healthcare, Pretoria 0184, South Africa; 12 Synergy Biomed Research Institute, East London 5201, South Africa; 13 South African Medical Research Council Vaccines and Infectious Diseases Analytics Research Unit, Faculty of Health Sciences, University of the Witwatersrand, Johannesburg 2195, South Africa; 14 Wits Infectious Diseases and Oncology Research Institute, Faculty of Health Sciences, University of the Witwatersrand, Johannesburg 2195, South Africa; 15 Worldwide Safety, Pfizer Srl, 20152 Milan, Italy; 16 Worldwide Safety, Pfizer Hellas SA, 55535 Thessaloniki, Greece

**Keywords:** clinical trial, HIV, immunogenicity, infants, LRTI, maternal vaccination, RSV, safety, transplacental transfer

## Abstract

**Background/Objectives:** HIV-exposed uninfected (HEU) infants experience increased severe respiratory syncytial virus lower respiratory tract illness (RSV-LRTI) rates compared with HIV-unexposed infants. Maternal bivalent RSVpreF vaccination can prevent infant RSV-LRTI but data from HEU infants are lacking. **Methods:** This phase 3 randomized, double-blinded trial assessed RSVpreF safety and immunogenicity in pregnant participants from South Africa living with HIV and their infants. Maternal participants with stable HIV disease taking antiretroviral therapy received RSVpreF or placebo (24–36 weeks’ gestation). Primary safety endpoints included reactogenicity through 7 days after vaccination (maternal participants), adverse events (AEs) through 1 month after vaccination (maternal participants) or birth (infants), and serious AEs (SAEs) throughout the study (maternal participants) or through 6 months after birth (infants). Immune responses were evaluated by 50% RSV-A and RSV-B neutralizing titers prevaccination and at delivery (maternal participants) or birth (infants). **Results:** Overall, 343 maternal participants received RSVpreF (*n* = 172) or placebo (*n* = 171). Most reactogenicity events were mild/moderate. AEs and SAEs were generally reported at similar frequencies in maternal RSVpreF and placebo groups including percentages of hypertensive disorders of pregnancy. There were no safety concerns in infants; percentages of reported AEs and SAEs were generally similar between RSVpreF and placebo groups and no difference in preterm birth. RSVpreF elicited high maternal neutralizing RSV-A and RSV-B immune responses, with efficient RSV antibody transplacental transfer to infants demonstrated by levels greater than the placebo group at birth (geometric mean ratios (GMRs) of RSVpreF to placebo were 7.8 for RSV-A and 6.8 for RSV-B) and by comparison with a cohort of HIV-unexposed infants from the pivotal phase 3 efficacy trial (GMRs of HEU to HIV-unexposed infants were 0.86 for RSV-A and 0.72 for RSV-B). **Conclusions:** These results support maternal RSVpreF vaccination among those living with stable HIV for preventing RSV-LRTI in HEU infants. (NCT06325657).

## 1. Introduction

More than 1 million pregnant individuals are estimated to be living with HIV globally, with the greatest burden in sub-Saharan Africa particularly in South Africa [1,2,3]. Worldwide, approximately 84% of pregnant individuals living with HIV received antiretroviral therapy (ART) to prevent vertical transmission, which has substantially reduced mother-to-child transmission of HIV [1,4,5,6,7]. Consequently, a large population of HIV-exposed but uninfected (HEU) children exists, particularly in sub-Saharan Africa [8], and even with ART, HEU infants experience increased rates and severity of childhood infections with associated morbidity and mortality when compared with HIV-unexposed infants [4,9,10,11].

Respiratory syncytial virus (RSV)-associated lower respiratory tract illness (RSV-LRTI) is a substantial cause of morbidity and mortality in young infants worldwide, with the greatest burden being outside of high-income regions [12]. Compared with pregnant individuals who are HIV-negative, pregnant individuals who are living with HIV experience higher incidences of RSV-associated illness during pregnancy and the postpartum period [13]. Impaired transplacental transfer of RSV-neutralizing antibodies and heightened transmission of RSV infection from the mother to the infant in the postpartum period likely contribute to the increased risk of severe RSV-LRTI in HEU infants, including higher hospitalization and mortality rates compared with HIV-unexposed uninfected infants [13,14,15,16]. The use of combination ART and management of hypergammaglobulinemia may mitigate these risks; however, data on the effectiveness of these approaches are limited, and economic and infrastructural barriers can impede access to these management approaches, particularly where they are needed most [14,15,17].

Maternal vaccination offers an attractive strategy for prevention of RSV illness in infants for several reasons. Vaccination during pregnancy can protect the infant against a number of diseases; infants can be protected in the susceptible window between birth and initiation of routine infant vaccines [18]. The phase 3 Maternal Immunization Study for Safety and Efficacy (MATISSE) clinical trial evaluated the efficacy and safety of maternal vaccination of healthy HIV-negative pregnant individuals with the bivalent RSV prefusion F (RSVpreF) vaccine in preventing RSV-LRTI in infants [19]. RSVpreF was well tolerated with no safety concerns in pregnant and infant participants and vaccine efficacy was 82% against severe RSV-LRTI in infants within 90 days after birth [19]. Additionally, RSVpreF elicited robust RSV-neutralizing responses in pregnant participants with their infants also achieving high neutralizing titers [20]. RSVpreF subsequently received licensure and is recommended in several countries and regions for use during pregnancy to protect infants against RSV-LRTI through 6 months of age [21,22,23,24,25,26,27].

With RSVpreF efficacy being demonstrated among infants born to healthy pregnant individuals [19], evaluating maternal vaccination with RSVpreF in pregnant individuals living with HIV and their infants is critical given the increased risk of serious RSV illness in HEU infants [28].

## 2. Methods

### 2.1. Design

The Maternal RSV Immunobridging Study (MORISOT) was a phase 3, multicenter, randomized, double-blinded, placebo-controlled clinical trial assessing the safety, tolerability, and immunogenicity of RSVpreF administered to pregnant participants living with HIV, as well as safety and immunogenicity in their infants (NCT06325657). Maternal participants, who had protocol-defined stable HIV disease (stable ART regimen for ≥90 days and documented HIV viral load < 1000 copies/mL and CD4 count >200 cells/mm^3^ within the 14 days before randomization) and an uncomplicated singleton pregnancy, received study intervention between 24 and 36 weeks of gestation. Gestational age was based on the date of the last menstrual period and a first or second trimester ultrasound as described in the Supplementary Materials. Maternal participants with previous pregnancy complications including previous preterm delivery (i.e., ≤34 weeks’ gestation), stillbirth, neonatal death, or previous infant with a known genetic disorder or significant congenital anomaly were excluded. Further eligibility criteria are provided in the Supplementary Materials.

The study complied with international consensus ethical principles, as well as applicable good clinical practice guidelines, laws, and regulations, as outlined in the Supplementary Materials. The protocol and other relevant documents were reviewed and approved by an institutional review board or ethics committee at the study sites before the study was initiated. Maternal participants (or their parent(s)/legal guardian if the maternal participant was a minor), were required to provide signed informed consent before study enrollment and before any study-related procedures were performed. Written informed consent from mothers (or their parent(s)/legal guardian for maternal participants who were minors) was also required for inclusion of their fetus during the pregnancy and for the infant’s continuation in the study after birth, as well as for obtaining umbilical cord blood.

### 2.2. Interventions

The active ingredients in the bivalent RSVpreF vaccine at the 120-µg dose level are stabilized RSV prefusion F antigens in equal amounts from RSV subgroup A (RSV-A; 60 µg) and RSV subgroup B (RSV-B; 60 µg). Maternal participants were randomized in a 1:1 ratio to receive a single dose of either RSVpreF or placebo (which was a lyophile match to RSVpreF), which were administered by intramuscular injection into the deltoid muscle, preferably of the nondominant arm.

### 2.3. Assessments and Endpoints

Safety assessments were the primary endpoints in maternal and infant participants. Maternal participants recorded a baseline assessment of prespecified systemic events (fatigue, headache, vomiting, nausea, diarrhea, muscle pain, and joint pain), including their severity (grading scale is provided in Table S1 [29]), in an electronic diary before receiving study intervention. Maternal participants also monitored and recorded these systemic events as well as prespecified local reactions (redness, swelling, and injection site pain) and oral temperature in the electronic diary each day beginning from the day of receiving randomized study intervention and for 7 days thereafter for unresolved reactions or events. Any reactogenicity events ongoing after this time were followed until resolution.

Adverse events (AEs) and serious AEs (SAEs) in both maternal and infant participants were categorized by terminology from version 28.0 of the Medical Dictionary for Regulatory Activities. AEs in maternal participants were recorded through 1 month after receipt of study intervention, and SAEs through the end of the study. Pregnancy outcomes collected at delivery among maternal participants included location, mode, and outcome of delivery, as well as the occurrence of any delivery complications. In infants, specific outcomes at birth (including gestational age, Apgar score, and infant outcomes (i.e., normal, congenital malformation or anomaly, other neonatal problem, or unknown)), AEs from birth through 1 month of age, and SAEs and newly diagnosed chronic medical conditions (NDCMCs) from birth through 6 months of age were recorded.

AEs of special interest (AESIs) for maternal participants were collected throughout the study and included preterm delivery (at <37 0/7 weeks’ gestation) and diagnoses of Guillain-Barré syndrome, acute polyneuropathy without an underlying etiology, hypertensive disorders of pregnancy, and atrial fibrillation as further described in the Supplementary Materials. AESIs for infant participants included preterm birth (born at <37 0/7 weeks’ gestation), birthweight of <2500 g, and developmental delay (which was assessed by clinical experts using local guidelines for standard of care). AESIs of extremely preterm birth (born at <28 0/7 weeks’ gestation) and extremely low birthweight (≤1000 g) were also reported as SAEs.

Maternal serum samples (before vaccination and at the time of delivery (preferably before delivery and at most within 48 h of delivery)) were collected for immunogenicity assessments. All infants had a cord blood sample collected from the umbilical cord vein at birth (if a cord blood sample was unavailable, a blood sample from the infant could be collected (preferably within 24 h after birth and within 7 days)). Maternal serum samples and infant cord blood samples were assayed for RSV-A and RSV-B serum neutralizing titers as described previously [30].

For maternal participants, the immune response was estimated by the 50% neutralizing geometric mean titer (GMT) before vaccination and at delivery and the geometric mean fold rise (GMFR) from before vaccination to the time of delivery for RSV-A, RSV-B, and combined RSV-A/B serum neutralizing titers, and the percentage of participants achieving seroresponse (i.e., ≥4-fold rise in serum neutralizing titers at delivery compared with prevaccination baseline or ≥4 times the lower limit of quantitation (LLOQ) if the baseline titer was below the LLOQ) as the secondary endpoint analyses, and geometric mean ratios (GMRs) of neutralizing GMTs at delivery (maternal participants) or birth (infant participants) from maternal participants living with HIV and their infants in the RSVpreF group compared to the placebo group. Post hoc and exploratory objectives included neutralizing GMTs at delivery (maternal participants) or birth (infant participants) from maternal participants living with HIV and their infants and the associated GMRs of neutralizing GMTs at delivery (maternal participants) or birth (infant participants) from maternal participants living with HIV and their infants compared with maternal South African participants without HIV and their infants from the MATISSE trial, respectively. For infant participants, functional antibody levels at birth were assessed as an exploratory endpoint and were estimated by the neutralizing GMTs and by the GMR among infants who were born to maternal participants who received RSVpreF compared with those in infants born to maternal participants who received placebo. A subgroup analysis assessed neutralizing GMTs and GMRs in infants at birth by gestational age at vaccination (i.e., 24–<28, 28–<32, and 32–36 weeks) with samples run contemporaneously.

A post hoc analysis modeled infants’ RSV-A/B combined 50% geometric mean neutralizing titers through 6 months of age (half-life: 42 days), comparing the MORISOT RSVpreF, MORISOT placebo, and MATISSE RSVpreF groups. Additional post hoc analyses calculated maternal and infant GMTs and GMRs (HIV to non-HIV) by maternal gestational age at vaccination, and maternal and infant GMTs and GMRs (RSVpreF to placebo) by maternal CD4 levels before vaccination and at delivery (maternal participants) and at birth (infants).

### 2.4. Statistics

This is a descriptive study; therefore, no statistical hypotheses were tested. A total of approximately 330 maternal participants were planned to be randomized (a target of approximately 165 maternal participants in each of the RSVpreF and placebo groups). Although the planned sample size was not based on any statistical hypothesis testing, the number enrolled was sufficient to characterize similarity between the current study and the MATISSE study to within a 2-fold margin (i.e., the lower bound of the 95% CI for the GMR between the RSVpreF recipients from MORISOT and the MATISSE South African subset would not be lower than 0.5).

Safety analyses were based on the safety populations. The maternal safety population included all randomized maternal participants who received at least 1 dose of study intervention. The infant safety population included all infants born to maternal participants who received study intervention. Descriptive statistics for categorical variables such as safety endpoints included the percentage, numerator, and denominator used in the percentage calculation. The associated exact 95% CIs for binary endpoints for each group were computed using the *F* distribution (i.e., the Clopper−Pearson method). The 95% CIs for the difference in the percentages (RSVpreF minus placebo), which were computed using the Miettinen and Nurminen method, were used to quantify the precision of the risk difference estimate and were not adjusted for multiplicity.

Immunogenicity endpoints in maternal participants were assessed in the maternal-evaluable immunogenicity population, which included all maternal participants who were eligible for the study, received the study intervention to which they were randomized, had blood drawn for assay testing within the specified time frame, had valid and determinate assay results for the proposed analysis, and had no major protocol violations. Immunogenicity endpoints in infant participants were assessed in the infant-evaluable immunogenicity population, which included all infant participants who were eligible for study participation, were born to maternal participants who received the study intervention to which they were randomized, had blood drawn for assay testing within the specified time frame, had valid and determinate assay results for the proposed analysis, and had no major protocol violations.

Continuous immunogenicity endpoints were logarithmically transformed for analysis. Geometric means and their associated 2-sided 95% CIs were derived by calculating group means and confidence limits on the natural log scale based on the Student *t* distribution; the results were then exponentiated. GMFRs for maternal participants with neutralizing titer values both before and after vaccination were calculated by determining the group mean of the difference in logarithmically transformed assay results with the earlier timepoint subtracted from the later timepoint, and then exponentiating the mean. The associated 2-sided 95% CIs for GMFRs were obtained by constructing confidence limits using the Student *t* distribution for the mean difference on the logarithm scale and exponentiating the confidence limits.

The GMRs were estimated by the ratio of the RSV-A, RSV-B, and RSV-A/B serum neutralizing titers at delivery (maternal participants) or birth (infant participants) from participants living with HIV from the current study compared with participants without HIV from South African sites in the phase 3 MATISSE study [20] (RSVpreF groups only). MATISSE participants were matched to the participants living with HIV in the current study by geographic location (i.e., from South Africa). The GMR was estimated from an analysis of covariance model, including adjustment for time from vaccination to delivery. The GMRs at delivery or birth were determined along with associated 2-sided 95% CIs. All MATISSE maternal participants from South Africa who received RSVpreF and were in the evaluable immunogenicity population, which included participants who received RSVpreF or placebo ≥14 days before delivery, were included in the model; blood samples were re-tested contemporaneously.

## 3. Results

### 3.1. Participants

This study was conducted at 14 sites in South Africa between 12 March 2024 and 11 June 2025 (see Supplementary File S1 for a listing of study sites). Of 549 pregnant individuals who were screened, 343 were randomized, of whom 172 received RSVpreF and 171 received placebo (Figure S1). All maternal participants apart from 2 placebo participants completed the delivery study visit, and 96.2% completed the study (RSVpreF, *n* = 167; placebo, *n* = 163). A total of 338 infants born to maternal participants were enrolled in the study (RSVpreF group, *n* = 171; placebo group, *n* = 167; Figure S2).

Maternal demographic characteristics were generally balanced in the RSVpreF and placebo groups (Table 1). The median age at vaccination was 31.0 (range, 16–44) years, and median gestational age at vaccination was 29.3 (range, 24.0–36.0) weeks. Demographic characteristics of the maternal participants living with HIV from the current study were generally balanced with the South African subset of maternal participants from the MATISSE trial who were included in the descriptive immunogenicity comparisons and vaccinations were well balanced by gestational age, with approximately one third of participants in both groups vaccinated at 24–28 weeks’ gestational age (Table S2).

### 3.2. Safety

Measures of HIV disease were generally balanced across the maternal RSVpreF and placebo groups (Table S3). The majority of maternal participants maintained an HIV viral load lower than the detectable testing limit from vaccination (RSVpreF, 82.0%; placebo, 81.8%) and through delivery (RSVpreF, 79.7%; placebo, 79.4%). The majority of maternal participants also had a CD4 count >500 cells/mm^3^ at vaccination (RSVpreF, 67.4%; placebo, 69.4%), while the percentage of participants with a CD4 count >500 cells/mm^3^ at delivery was 52.9% in the RSVpreF group and 47.1% in the placebo group. At delivery, 7 maternal participants had an HIV viral load of >1000 copies/mL (RSVpreF, *n* = 5 (2.9%); placebo, *n* = 2 (1.2%)).

Because of procedural noncompliance, electronic diary data for 11 participants from a single site were excluded from the reactogenicity analyses. It was reported that at this site, reactogenicity data for the 11 participants were entered on behalf of participants by a study site staff member, but the origin and accuracy of the entered electronic diary data could not be verified. All other study data associated with these participants were included in the analyses. A sensitivity analysis found that exclusion of this site did not affect the overall reactogenicity conclusions, supporting exclusion of the data from the reported analysis.

**Figure 1:**
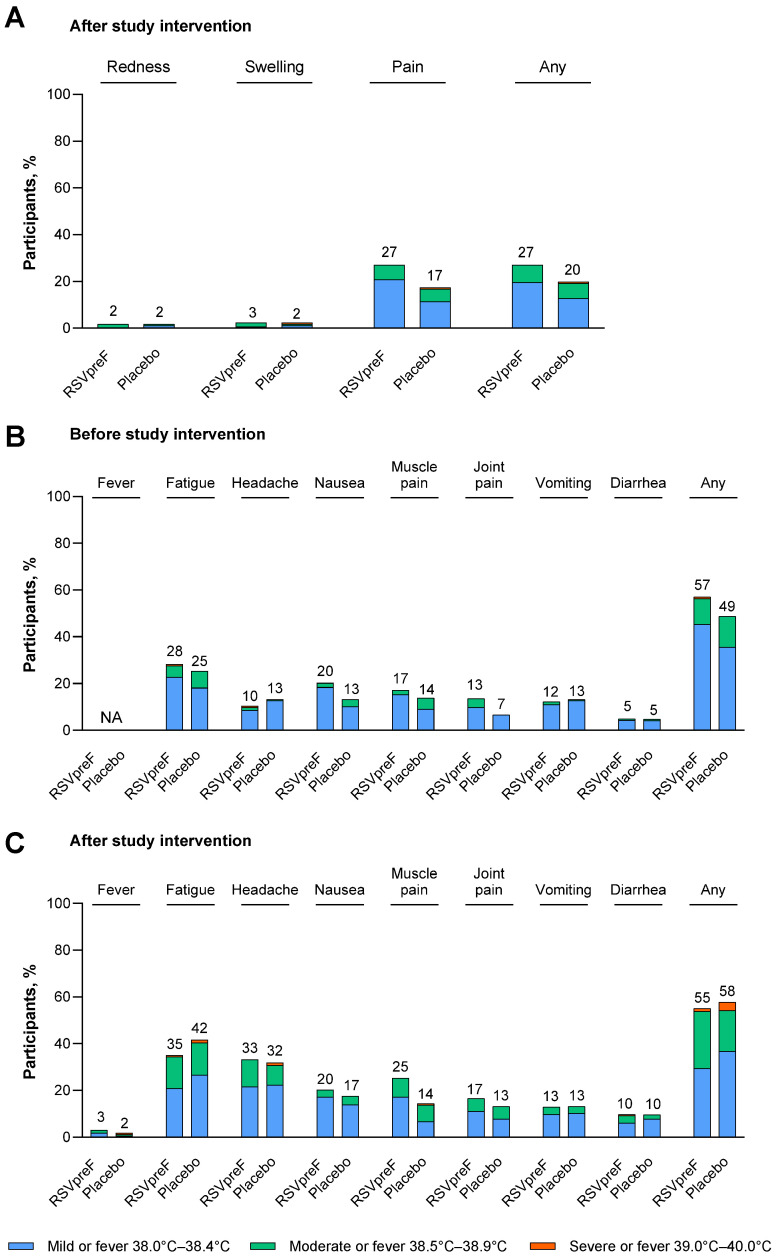
(**A**) Local reactions after study intervention, (**B**) systemic events before study intervention, and (**C**) systemic events after study intervention in maternal participants. Data are for the safety population of maternal participants who received RSVpreF excluding participants with a deviation at a single study site (N = 163) or placebo (N = 166). Local reactions and systemic events were prespecified and collected from Day 1 through Day 7 after vaccination. Systemic events (except fever) were also collected within 7 days before vaccination. Any includes any local reaction or any systemic event (excluding fever). The values above the bars are the percentage of maternal participants with that reaction or event. NA, not applicable.

Local reactions reported by maternal participants in the electronic diary within 7 days after receipt of study intervention are shown in Figure 1A. The most common local reaction was injection site pain, which was reported by 27.0% of RSVpreF recipients and 17.5% of placebo recipients. Nearly all local reactions were mild or moderate in severity. One participant who received placebo experienced severe injection site pain and swelling; there were no severe local reactions reported in maternal participants who received RSVpreF. The median onset of local reactions was 1 to 3 days after receipt of study intervention, and the median duration of local reactions was 1 to 2 days across all maternal participants.

The percentages of maternal participants reporting any AEs within 1 month after receipt of study intervention were 14.5% (*n* = 25) in the RSVpreF group and 24.1% (*n* = 41) in the placebo group; the majority of AEs were mild or moderate in severity (Figure S3A). Through 1 month after receipt of study intervention, 3 participants reported AEs that were considered by the investigator to be related to the study intervention (2 participants in the RSVpreF group (gestational hypertension and dizziness, each in 1 participant) and 1 participant with arthralgia in the placebo group). From vaccination throughout the duration of the study, SAEs were reported in 34.9% (*n* = 60) of RSVpreF recipients and 33.5% (*n* = 57) of placebo recipients, most commonly in the pregnancy, puerperium, and perinatal conditions system organ class (RSVpreF, 31.4% (*n* = 54); placebo, 30.0% (*n* = 51); Table S4). No SAEs were considered to be related to the study intervention by the investigator. Stillbirth was reported in 0.6% (*n* = 1) of RSVpreF recipients and 1.2% (*n* = 2) of placebo recipients.

**Figure 2:**
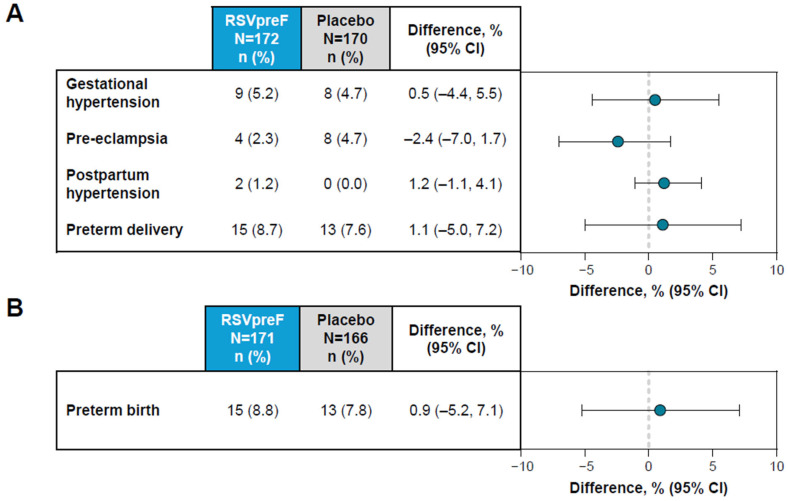
Adverse events of special interest in (**A**) maternal and (**B**) infant participants. Shown is the difference in proportions expressed as a percentage (RSVpreF minus placebo) with 2-sided 95% CIs based on the Miettinen and Nurminen method.

In maternal participants, the AESIs of hypertensive disorders of pregnancy (i.e., gestational hypertension (RSVpreF, 5.2%; placebo, 4.7%); and pre-eclampsia (RSVpreF, 2.3%; placebo, 4.7%)) and postpartum hypertension (RSVpreF, 1.2%; placebo, 0.0%) and preterm delivery (RSVpreF, 8.7%; placebo, 7.6%) occurred in a similar percentage of RSVpreF and placebo maternal recipients (Figure 2A). The risk difference estimates of the comparison between RSVpreF and placebo were between −2.4% (95% CI, −7.0%, 1.7%) and 1.2% (95% CI, −1.1%, 4.1%), with 95% CIs that crossed zero. No cases of eclampsia, Hemolysis, Elevated Liver enzymes and Low Platelets (HELLP) syndrome, atrial fibrillation or Guillain-Barré syndrome were reported.

The characteristics of maternal participants with hypertension, gestational hypertension, and pre-eclampsia are summarized in Table S5. Overall, 66.7% (*n* = 18/27) of participants with postpartum hypertension, gestational hypertension, or pre-eclampsia delivered at 37–<42 weeks’ gestational age (RSVpreF, 75.0% (9/12); placebo, 60.0% (9/15)), and the relative days of diagnosis from study intervention were >30 days for 66.7% (18/27) of maternal participants (RSVpreF, 66.7% (8/12); placebo, 66.7% (10/15)). The characteristics of maternal participants with preterm (RSVpreF, 15/171; placebo, 13/166) and term deliveries (RSVpreF, 156/171; placebo, 153/166) are summarized in Table S6. Most preterm deliveries were late preterm (i.e., 34–<37 weeks) in both groups (RSVpreF, 93.3% (14/15); placebo, 76.9%, (10/13)).

Through 1 month after birth, AEs in infant participants were reported in 34.5% (*n* = 59) and 44.0% (*n* = 73) of those infants born to maternal participants who received RSVpreF and placebo, respectively (Figure S3B). Most AEs were mild or moderate in severity and no AEs were reported as related to maternal study intervention. Through 1 month after birth, NDCMCs were reported in <1% of infant participants in either group. One infant in each group tested positive for HIV (an infant in the placebo group from birth to 1 month of age, and an infant in the RSVpreF group between 1 and 6 months of age). Throughout the study, SAEs occurred in 18.7% (*n* = 32) and 21.7% (*n* = 36) of infants from the RSVpreF and placebo groups, respectively.

In infants, the AESI of preterm birth was reported in 8.8% (*n* = 15) of infants born to maternal participants who received RSVpreF and in 7.8% (*n* = 13) of infants born to maternal participants who received placebo (Figure 2B). The risk difference estimate of the comparison between RSVpreF and placebo was 0.9% (95% CI, −5.2%, 7.1%), which equates to a relative risk of 1.12 (95% CI, 0.55, 2.28). The AESI of low birth weight was reported in 13.5% (*n* = 23) of infants in the RSVpreF group and 12.7% (*n* = 21) of infants in the placebo group, equating to a relative risk of 1.06 (95% CI, 0.61, 1.85). Corresponding values for the AESI of developmental delay within 6 months of birth were 0% for RSVpreF and 0.6% (*n* = 1) for placebo.

### 3.3. Immunogenicity

**Figure 3:**
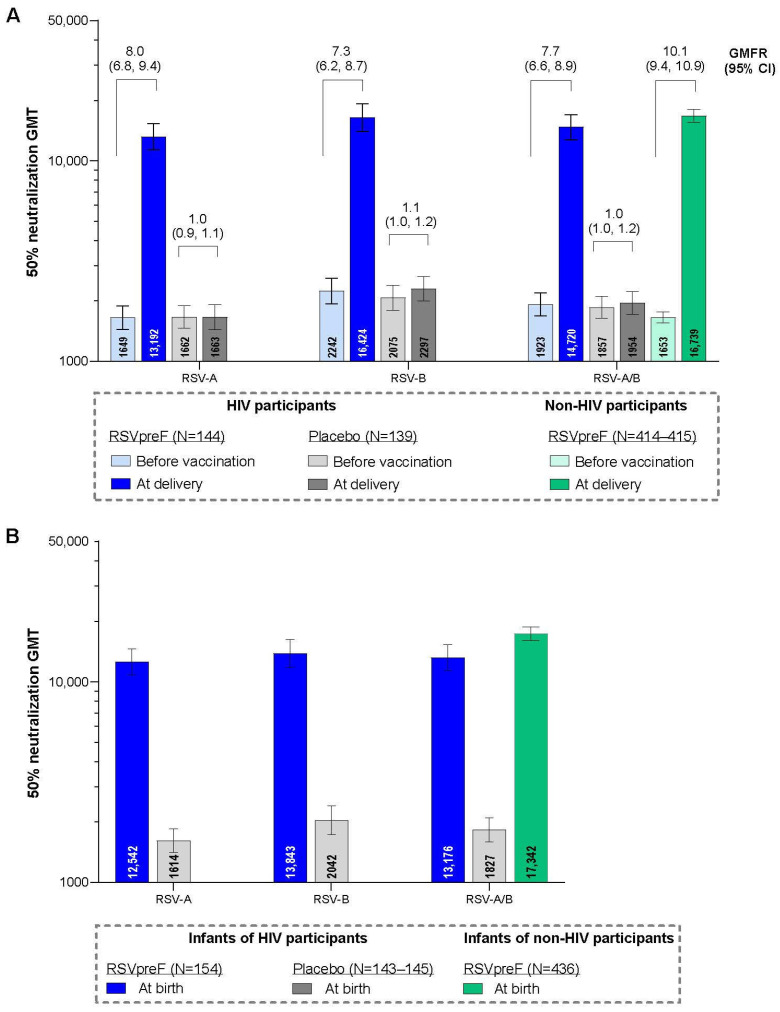
RSV-neutralizing titer GMTs and GMFRs in maternal participants (**A**) and GMTs in infant participants (**B**) with 95% CIs. Data are for the evaluable immunogenicity populations. LLOQ values were 242 for RSV-A and 99 for RSV-B neutralizing titers. Assay results below the LLOQ were set to 0.5 × LLOQ. GMTs and GMFRs were calculated by exponentiating the mean logarithm of the titers or the mean logarithm of the fold rises, respectively, with corresponding CIs based on the Student *t* distribution. For each individual, combined RSV-A/RSV-B was calculated as the geometric mean of titer or fold rise of RSV-A and RSV-B at the specified timepoint. The data for the non-HIV participants are from the South African participants from the MATISSE trial in a post hoc analysis. GMFR, geometric mean fold rise; GMT, geometric mean titer; LLOQ, lower limit of quantitation; N, number of participants with valid and determinate assay results.

**Figure 4:**
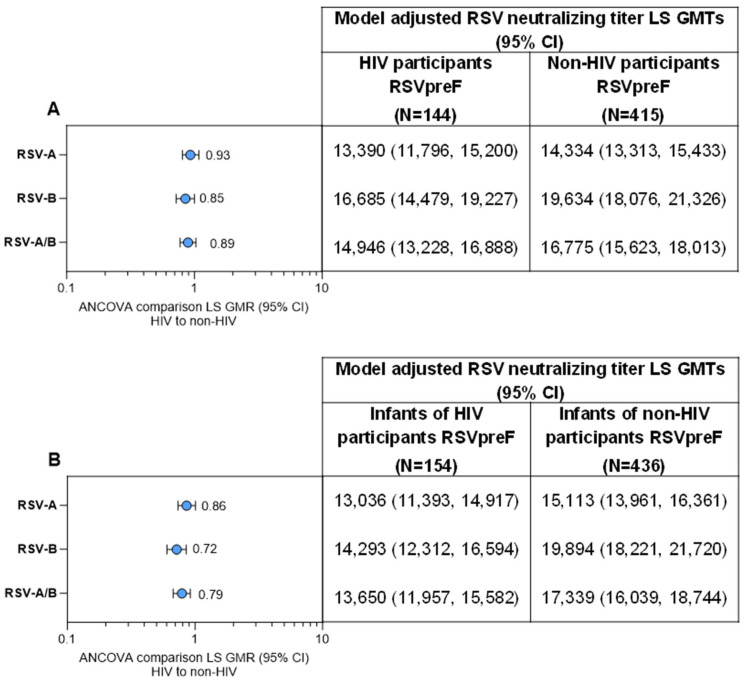
RSV-neutralizing titer GMTs and GMRs (**A**) at delivery in maternal participants living with HIV compared with maternal participants without HIV from the MATISSE trial and (**B**) at birth for the respective infant participants. Data are for the evaluable immunogenicity populations. The LLOQ values were 242 for RSV-A and 99 for RSV-B neutralizing titers. Assay results below the LLOQ were set to 0.5 × LLOQ. GMTs and GMRs and associated 2-sided CIs were calculated by exponentiating the LS means, respectively, and the corresponding CIs based on analysis of log-transformed titers using a regression model with population groups (HIV participants versus non-HIV participants) and maternal vaccination-to-delivery interval as covariates. For each individual, combined RSV-A/RSV-B was calculated as the geometric mean of titer or fold rise of RSV-A and RSV-B at the specified timepoint. ANCOVA, analysis of covariance; GMR, geometric mean ratio; GMT, geometric mean titer; LLOQ, lower limit of quantitation; LS, least square.

Neutralizing GMTs for RSV-A, RSV-B, and RSV-A/B increased substantially from before RSVpreF vaccination to after vaccination at delivery for maternal participants (Figure 3A). GMFRs (95% CI) from before vaccination to delivery were 8.0 (6.8, 9.4) for RSV-A, 7.3 (6.2, 8.7) for RSV-B, and 7.7 (6.6, 8.9) for RSV-A/B. Corresponding GMFRs for placebo recipients were 1.0 (0.9, 1.1), 1.1 (1.0, 1.2), and 1.0 (1.0, 1.2). In the post hoc analysis, the GMFR for RSV-A/B from before vaccination to delivery for matched maternal participants without HIV from the MATISSE trial was 10.1 (9.4, 10.9). Maternal GMRs for RSVpreF to placebo were approximately 1 before receipt of study intervention and >7 at delivery for RSV-A, RSV-B, and RSV-A/B (Table S7). Among those vaccinated with RSVpreF, GMRs (95% CIs) of RSV-neutralizing titers for maternal participants living with HIV compared with matched maternal participants without HIV from the MATISSE trial were 0.93 (0.81, 1.08), 0.85 (0.72, 1.00), and 0.89 (0.77, 1.03) for RSV-A, RSV-B, and RSV-A/B, respectively (Figure 4A).

The percentages of maternal participants receiving RSVpreF achieving a seroresponse were 76.4%, 73.6%, and 78.5% for RSV-A, RSV-B, and RSV-A/B (Figure S4). Corresponding values for placebo were ≤5%.

Among the immunogenicity evaluable population, infants born to maternal participants who received RSVpreF during pregnancy had substantially higher neutralizing GMTs at birth for RSV-A, RSV-B, and RSV-A/B (12,542, 13,843, and 13,176, respectively) compared with infants born to maternal participants in the placebo group (1614, 2042, and 1827; Figure 3B). In the post hoc analysis, the neutralizing RSV-A/B GMT at birth for infants born to maternal participants without HIV from the MATISSE trial was 17,342. Infant GMRs at delivery for RSVpreF to placebo were ≥6.8 for RSV-A, RSV-B, and RSV-A/B (Table S7). GMRs (95% CI) for infants born to maternal participants living with HIV compared with infants born to maternal participants without HIV from the MATISSE trial were 0.86 (0.74, 1.01), 0.72 (0.60, 0.85), and 0.79 (0.68, 0. 92) for RSV-A, RSV-B, and RSV-A/B (Figure 4B). A subgroup analysis of infant neutralizing GMTs and GMRs (RSVpreF vs. placebo) at birth by maternal gestational age group at vaccination (i.e., 24–<28, 28–<32, and 32−36 weeks) is provided in Table S8. Infants born to mothers vaccinated with RSVpreF had higher RSV-neutralizing titers at birth compared to those born to mothers who received placebo across all gestational age groups; GMRs were higher in the 28–<32 and 32–36 weeks’ gestational age groups compared to the 24–<28 weeks’ gestational age group.

Modeled infant combined RSV-A/B neutralizing titers through 6 months of age showed a consistent decline in titers across all groups over 6 months (Figure S5). When stratified by gestational age at vaccination, unadjusted GMRs (95% CI) for HIV to non-HIV participants at delivery for RSV-A/B combined were 0.87 (0.68, 1.11), 0.79 (0.61, 1.03), and 0.90 (0.70, 1.15) for participants vaccinated at 24–<28 weeks, 28–<32 weeks, and 32–≤36 weeks gestational age, respectively (Table S9). Corresponding unadjusted infant GMRs (95% CI) at birth were 0.65 (0.50, 0.85), 0.82 (0.62, 1.10), and 0.80 (0.62, 1.04), respectively (Table S10). When stratified by maternal CD4 levels, unadjusted GMRs (95% CI) for RSVpreF to placebo at delivery for RSV-A/B combined were 8.2 (6.61, 10.17) for participants with CD4 counts >500 cells/mm^3^ and 5.80 (3.72, 9.02) for those with CD4 counts ≤500 cells/mm^3^ (Table S11). Corresponding unadjusted infant GMRs (95% CI) at birth were 7.94 (6.40, 9.84) and 5.26 (3.24, 8.56), respectively (Table S12).

## 4. Discussion

Maternal RSV vaccination is an important strategy for protecting pregnant individuals living with HIV and their infants, which are populations at increased risk of severe RSV disease [31,32,33,34]. This is particularly relevant in South Africa, where the current MORISOT study was conducted, which has one of the largest populations of individuals living with HIV globally (an estimated 7.7 million adults ≥15 years of age in 2024) and where nationally, as of 2022, more than one quarter of pregnant individuals attending antenatal care were estimated to be HIV-positive [35,36,37]. In South Africa, HEU infants younger than 6 months experience an estimated 1.4-fold higher annual incidence of LRTI-associated hospitalization and those 29 days to 12 months of age have an adjusted incidence rate ratio of 2.8 for infectious-cause hospitalization when compared with HIV-unexposed infants of the same age groups [16,38]. Compared with unexposed infants, hospitalized HEU infants also had longer lengths of hospital stay, greater frequency of mechanical ventilation use, and higher in-hospital mortality [16].

In the current MORISOT study, maternal vaccination with RSVpreF had an acceptable safety and tolerability profile in pregnant participants living with HIV. For maternal participants, local reactions and systemic events were generally mild to moderate in severity and similar when comparing RSVpreF and placebo groups, except for injection site pain and muscle pain, which were both more commonly reported in the RSVpreF group. The percentages of participants reporting local reactions and systemic events after RSVpreF were generally similar or slightly lower than those reported in the MATISSE trial [19]. AEs and SAEs were generally reported at similar frequencies in the maternal RSVpreF and placebo groups, which is consistent with previous observations in maternal vaccination trials with RSVpreF in pregnant individuals [19,20,39].

AESIs observed in maternal participants in the current MORISOT trial included hypertensive disorders of pregnancy and preterm delivery and were observed with similar frequency in the RSVpreF and placebo groups. Although the study was not powered to detect a difference in preterm delivery in pregnant individuals living with HIV, this is a notable distinction from the MATISSE study in which a post hoc analysis by country noted an imbalance in preterm births among infants in the RSVpreF group in South Africa (RSVpreF, 8.3%; placebo, 4.0% (relative risk, 2.06: 95% CI, 1.21, 3.51)), despite no increase in preterm birth among infants in the RSVpreF group (5.7%) versus the placebo group (4.7%) for the study as a whole (relative risk, 1.20; 95% CI, 0.98, 1.46) [40]. Additionally, in the MATISSE study, hypertensive disorders of pregnancy were observed in a similar percentage of RSVpreF recipients overall compared with placebo recipients (≤1% for any preferred term encompassing hypertensive disorders of pregnancy in either group), but in a higher percentage of RSVpreF recipients who delivered preterm, which was mostly driven by pre-eclampsia (14.6% (30/206) of participants in the RSVpreF group and 9.9% (17/172) of participants in the placebo group) [20,40]. The overall frequencies of preterm delivery and hypertensive disorders of pregnancy were generally higher in MORISOT relative to participants enrolled in South Africa from MATISSE; this is not unexpected, given the elevated risk of preterm delivery and hypertensive disorders of pregnancy in pregnant people living with HIV, relative to those without [41,42,43,44,45,46,47,48,49,50]. Given the difference in sample size and participant risk profile, there is limited ability to compare the two studies and draw conclusions regarding preterm delivery and hypertensive disorders of pregnancy. However, preliminary data from real-world studies continue to support that there are no observed increases in preterm deliveries in individuals after RSVpreF vaccination compared with individuals who do not receive RSVpreF, while real-world evidence regarding risk of hypertensive disorders in pregnancy and receipt of RSVpreF are ongoing [51,52,53].

There were no new safety concerns in infant participants; percentages of infants with AEs, SAEs, NDCMCs, and AESIs were generally similar between RSVpreF and placebo groups. The AESI observed in infant participants included preterm birth and low birth weight, which were balanced across the infants delivered by RSVpreF and placebo recipients.

These encouraging safety results for RSVpreF contribute to the evidence of the benefit of maternal RSV vaccination in individuals living with HIV, which also includes safety analyses of influenza, pneumococcal, and investigational GBS vaccines that found no difference in vaccine safety in pregnant individuals living with HIV compared with pregnant individuals without HIV [32].

Maternal vaccination in pregnant individuals utilizes the natural transplacental transfer of maternal antibodies to the fetus, and passive protection of infants in the vulnerable first months of life [54]. In this study, RSVpreF elicited maternal neutralizing RSV-A and RSV-B immune responses in pregnant individuals living with HIV, with efficient transplacental transfer of RSV antibodies to infants at birth. In a descriptive comparison with pregnant participants without HIV from South African study sites from the MATISSE trial, neutralizing RSV-A and RSV-B titers were slightly lower among maternal participants living with HIV and their infants, but substantially higher than those from MORISOT placebo recipients and their infants. In previous vaccine and infection studies, pregnant individuals living with HIV demonstrated reduced transplacental antibody transfer after vaccination and after natural infection [55,56,57,58,59]. Mechanisms linking HIV infection and reduced transplacental antibody transfer are likely multifactorial including lower maternal antibody concentrations, hypergammaglobulinemia, and placental insufficiency [57,58,59]. Therefore, it is encouraging that maternal RSVpreF vaccination resulted in infant neutralizing GMTs substantially higher than with placebo and higher than a serum palivizumab concentration previously associated with nearly complete protection against RSV-associated pediatric intensive care unit admission [20,60].

Study strengths include the randomized, placebo-controlled study design in only pregnant individuals living with HIV. Enrolled maternal participants were established on ART with mostly undetectable HIV viral load and robust CD4 counts. Given the improved access to perinatal ART globally [1,61], the study findings can inform much of the pregnant population living with HIV but are not generalizable to all individuals living with HIV, particularly those newly diagnosed, without access to ART or with poorly controlled HIV infection. Limitations include the descriptive nature of the immunogenicity comparison; the study was not powered for formal hypothesis testing of a comparison to participants from the MATISSE trial. Additionally, the 2 trials were not contemporaneous, with the MATISSE trial conducted from 2020 to 2022, during the COVID-19 pandemic. Nevertheless, serum samples from both trials were assayed at the same time for this analysis. Study participants were only from South Africa, possibly limiting generalizability to other regions, although there is little evidence of geographic heterogeneity in RSV vaccine responses generally [20]. Finally, although immunogenicity was described relative to participants in the pivotal MATISSE study, efficacy of RSVpreF for protection against RSV-LRTI in infants was not assessed in MORISOT.

## 5. Conclusions

RSVpreF was well tolerated with an acceptable safety profile in pregnant individuals living with HIV and in their infants. RSVpreF elicited robust immune responses in these pregnant individuals, with efficient transplacental transfer of antibodies observed in their infants. These results support the use of RSVpreF during pregnancy among those living with HIV for the prevention of RSV-associated LRTI in their infants and contribute to the growing evidence of favorable safety outcomes of RSVpreF in the pregnant population.

## Figures and Tables

**Table 1:** Demographics of maternal and infant participants.

Characteristic	RSVpreF	Placebo	Total
**Maternal demographic**	**(N = 172)**	**(N = 170)**	**(N = 342)**
Age at vaccination, years			
Mean (SD)	31.3 (5.81)	31.0 (6.01)	31.1 (5.91)
Median (range)	31.0 (19–44)	31.0 (16–43)	31.0 (16–44)
Gestational age at vaccination, weeks			
Mean (SD)	29.5 (3.76)	29.4 (3.70)	29.5 (3.72)
Median (range)	29.30 (24.0–36.0)	29.10 (24.0–36.0)	29.30 (24.0–36.0)
Race, *n* (%)			
Black	171 (99.4)	169 (99.4)	340 (99.4)
Not reported	1 (0.6)	1 (0.6)	2 (0.6)
**Infant demographic and live birth outcome**	**(N = 171)**	**(N = 166)**	**(N = 337)**
Sex, *n* (%)			
Male	91 (53.2)	91 (54.8)	182 (54.0)
Female	80 (46.8)	75 (45.2)	155 (46.0)
Race, *n* (%)			
Black	170 (99.4)	166 (100.0)	336 (99.7)
Not reported	1 (0.6)	0	1 (0.3)

SD, standard deviation. Data are for the maternal and infant safety populations.

## Data Availability

Upon request, and subject to review, Pfizer will provide the data that support the findings of this study. Subject to certain criteria, conditions, and exceptions, Pfizer may also provide access to the related individual de-identified participant data. See https://www.pfizer.com/science/clinical-trials/trial-data-and-results for more information (accessed on 11 November 2025).

## Supplementary Materials

The following supporting information can be downloaded at: https://www.mdpi.com/article/10.3390/vaccines13121218/s1, Supplementary File S1; Figure S1. Disposition of maternal participants; Figure S2. Disposition of infant participants; Figure S3. Summary of adverse events (95% CIs) and deaths in (A) maternal participations and (B) infant participants; Figure S4. Maternal participants achieving RSV neutralizing titer seroresponse at delivery; Figure S5. Modeled infant combined RSV-A/RSV-B neutralizing titers through 6 months of age based on half-life of 42 days from infants of maternal participants living with HIV compared with infants of maternal participants from the MATISSE trial; Table S1. Severity grading of local reactions, systemic events, and fever; Table S2. Demographics for maternal participants living with HIV (current study) and non-HIV participants (MATISSE). Table S3. HIV viral load and CD4 count for maternal participants at vaccination and at delivery; Table S4. Serious adverse events in maternal recipients (reported from vaccination throughout the study) and for infant participants) reported through 1 month and 6 months after birth) by system organ class and preferred term; Table S5. Characteristics of maternal participants with hypertension and hypertensive disorders of pregnancy; Table S6. Characteristics of maternal participants with preterm and term delivery; Table S7. RSV neutralizing titer GMRs before vaccination and at delivery (maternal participants) and birth (infant participants); Table S8. Infant RSV neutralizing GMTs and GMRs at birth by maternal gestational age at vaccination; Table S9. Maternal unadjusted RSV neutralizing GMTs and GMRs by gestational age at vaccination; Table S10. Infant unadjusted RSV neutralizing GMTs and GMRs at birth by maternal gestational age at vaccination; Table S11. Maternal unadjusted RSV neutralizing GMTs and GMRs by maternal CD4 levels; Table S12. Infant unadjusted RSV neutralizing GMTs and GMRs at birth by maternal CD4 levels
