# Supplementary material for: Safety, Tolerability, and Immunogenicity of RSVpreF Vaccine in Pregnant Individuals Living with HIV
Source: Vaccines (Basel). 2025 Dec 1;13(12):1218. doi: 10.3390/vaccines13121218 (PMC12737651; doi:10.3390/vaccines13121218)

Figure S3. Summary of adverse events (95% CIs) and deaths in (A) maternal participations* and (B) infant participants†

Data are for the safety population. *In maternal participants, data are presented through 1 month after study intervention, except for serious AEs, AEs leading to withdrawal, AESIs, and deaths, which are shown throughout the study. †In infant participants, data are presented through 1 month after birth except for serious AEs, AEs leading to withdrawal, NDCMCs, AESIs, and deaths, which are shown throughout the study. AESI, adverse event of special interest; NDCMC, newly diagnosed chronic medical condition.

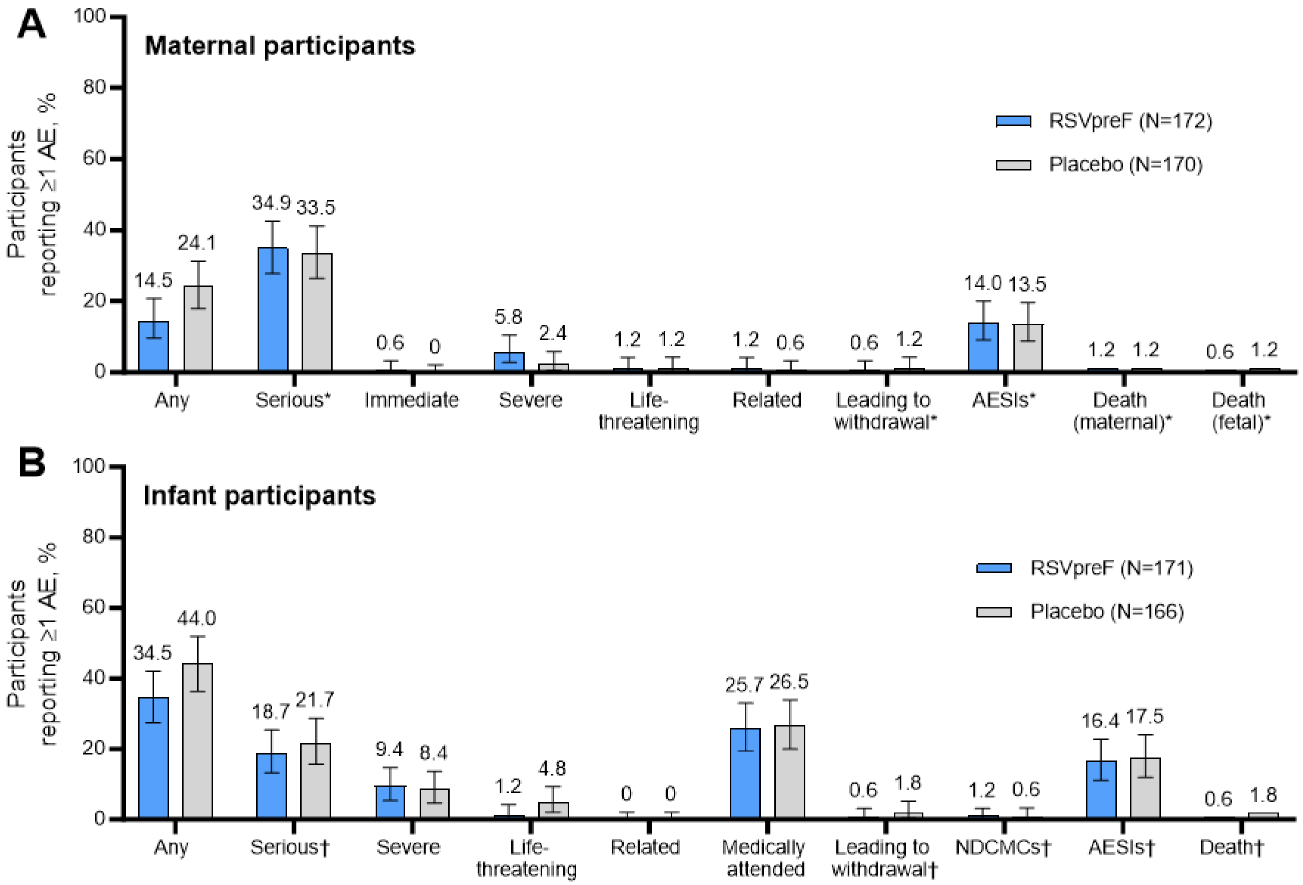

Supplement: Supplementary file 1 [file vaccines-13-01218-s001.zip › Figure S3.pdf]
