# Supplementary material for: Safety, Tolerability, and Immunogenicity of RSVpreF Vaccine in Pregnant Individuals Living with HIV
Source: Vaccines (Basel). 2025 Dec 1;13(12):1218. doi: 10.3390/vaccines13121218 (PMC12737651; doi:10.3390/vaccines13121218)

Figure S4. Maternal participants achieving RSV neutralizing titer seroresponse at delivery

Data are for the evaluable immunogenicity population. Seroresponse was defined as achieving a ≥4-fold rise from baseline if the baseline measurement was >LLOQ. If the baseline measurement was <LLOQ, a postvaccination assay result ≥4 × LLOQ was considered a seroresponse. The LLOQ values were 242 for RSV-A and 99 for RSV-B neutralizing titers. Assay results below the LLOQ were set to 0.5 × LLOQ. Lower limit of quantitation; RSV, respiratory syncytial virus.

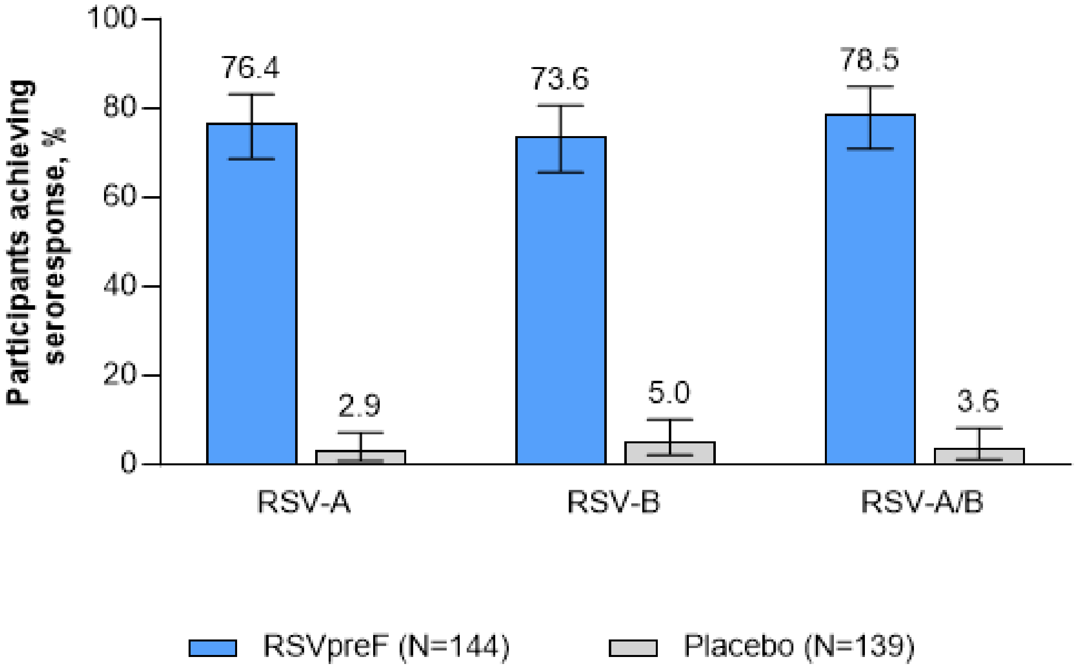

Supplement: Supplementary file 1 [file vaccines-13-01218-s001.zip › Figure S4.pdf]
