# Supplementary material for: Safety, Tolerability, and Immunogenicity of RSVpreF Vaccine in Pregnant Individuals Living with HIV
Source: Vaccines (Basel). 2025 Dec 1;13(12):1218. doi: 10.3390/vaccines13121218 (PMC12737651; doi:10.3390/vaccines13121218)

Figure S5. Modeled infant combined RSV-A/RSV-B neutralizing titers through 6 months of age based on half-life of 42 days from infants of maternal participants living with HIV compared with infants of maternal participants from the MATISSE trial

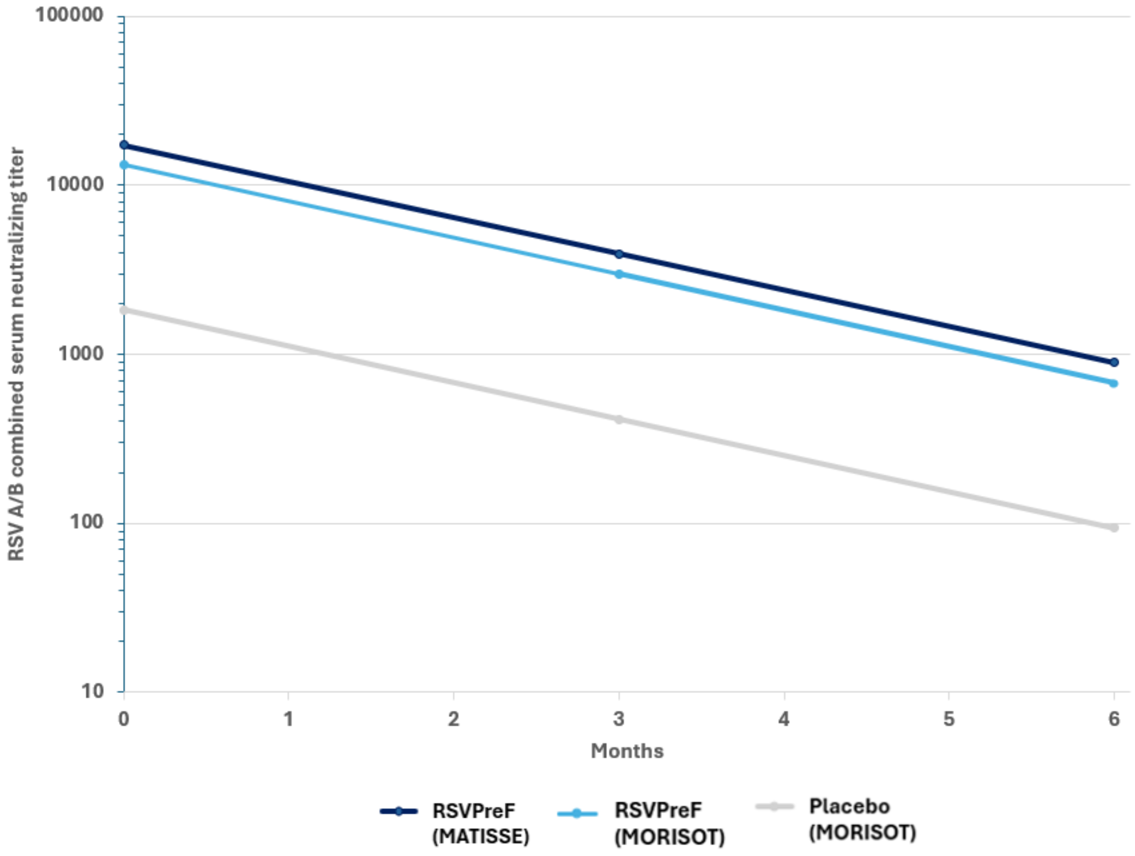

Supplement: Supplementary file 1 [file vaccines-13-01218-s001.zip › Figure S5.pdf]
