# Supplementary material for: Safety, Tolerability, and Immunogenicity of RSVpreF Vaccine in Pregnant Individuals Living with HIV
Source: Vaccines (Basel). 2025 Dec 1;13(12):1218. doi: 10.3390/vaccines13121218 (PMC12737651; doi:10.3390/vaccines13121218)
Supplement: Supplementary file 1 [file vaccines-13-01218-s001.zip › Supplementary File S1.pdf]

SUPPLEMENTARY MATERIALS

SUPPLEMENTARY FILE S1

Eligibility criteria for maternal participants

Inclusion criteria

Where study procedures were used to determine eligibility criteria (eg, ultrasound examination, syphilis testing, hepatitis B virus [HBV] testing, screening for active tuberculosis [TB] as per local guidelines, HIV viral load and CD4 count), and were not performed as part of routine prenatal care, they must have been performed as part of this clinical trial and reviewed by the investigator or a qualified designee before randomization.

HIV viral load and CD4 count must have been assessed from samples obtained within 14 days before randomization. Screening and testing for syphilis, active TB, and HBV must have been assessed during this pregnancy and before randomization. Each maternal participant must have provided informed consent for themselves and the unborn infant before performing any procedures, including those that were being done exclusively to meet study eligibility criteria, per all applicable requirements.

<u>Age and gestational age</u>

- Maternal participants ≤49 years of age between 24 0/7 and 36 0/7 weeks of gestation on the day of planned vaccination, with an uncomplicated, singleton pregnancy and with no known increased risk for complications. The gestational age (GA) must have been based on 1 of the following composite criteria which were determined by the timing and availability of data on the last menstrual period (LMP) and an ultrasound examination performed in the first or second trimester. The earliest ultrasound data available during the current pregnancy was used. Where the routine practice was for the GA determination to be based on the first-trimester ultrasound examination alone, without the LMP, this routine practice was accepted.
    - First-trimester data available (data obtained at ≤13 6/7 weeks):
        - The date of the first day of the reported LMP could be used to establish the GA if corroborated by a first-trimester ultrasound examination.
        - If there was a discrepancy of >5 days between the LMP-determined GA and an ultrasound result at ≤8 6/7 weeks or the LMP was uncertain/unknown, then the GA was to be determined using the first-trimester ultrasound result.
        - If there was a discrepancy of >7 days between the LMP-determined GA and an ultrasound result at 9 0/7 to 13 6/7 weeks or the LMP was uncertain/unknown, then the GA was determined using the first-trimester ultrasound result.
    - Second-trimester data available (data obtained at 14 0/7 to 27 6/7 weeks):
        - The date of the first day of the reported LMP could be used to establish the GA if corroborated by a second-trimester ultrasound result.
        - If there was a discrepancy of >7 days between the LMP-determined GA and the ultrasound result at 14 0/7 to 15 6/7 weeks or if the LMP was uncertain/unknown, then the GA was determined using the second-trimester ultrasound result.

    - If there was a discrepancy of >10 days between the LMP-determined GA and the ultrasound result at 16 0/7 to 21 6/7 weeks or if the LMP was uncertain/unknown, then the GA was determined using the second-trimester ultrasound result.

    - If there was a discrepancy of >14 days between the LMP-determined GA and the ultrasound result at 22 0/7 to 27 6/7 weeks or if the LMP was uncertain/unknown, then the GA was determined using the second-trimester ultrasound result.

Disease characteristics

- Confirmed stable HIV disease, defined as a documented HIV viral load <1000 copies/mL and CD4 count >200 cells/mm^3, within 14 days before randomization.
- Current and stable use of antiretroviral therapy for ≥90 days before randomization.
- Receiving prenatal standard of care based on country requirements.
- Had a fetal anomaly ultrasound examination performed at ≥18 weeks of pregnancy with no significant fetal abnormalities observed.
- Determined by medical history, physical examination, and clinical judgment to be appropriate for inclusion in the study.
- Within 14 days before randomization documented negative syphilis and negative HBV surface antigen tests, and absence of active TB infection demonstrated by a negative active TB screening performed per local guideline and no current or previous use of anti-TB treatments during this pregnancy.

Other inclusion criteria

- Willing to give informed consent for the participant's infant to participate in the study.
- Intention to deliver at a hospital or birthing facility where study procedures could be obtained.

Exclusion criteria

Medical conditions

- Any medical or psychiatric condition, including recent (within the past year) or active suicidal ideation/behavior or laboratory abnormality that could increase the risk of study participation.
- Prepregnancy body mass index (BMI) of >40 kg/m^2. If pre-pregnancy BMI was not available, the BMI at the time of the first obstetric visit during the current pregnancy was used.
- Participant with opportunistic infections or malignancy.
- History of active chronic viral hepatitis with biochemical evidence of aspartate aminotransferase or alanine aminotransferase values >5 times the upper limit of normal within 6 months before enrollment.
- Bleeding diathesis or condition associated with prolonged bleeding that contraindicated intramuscular injection.
- History of severe adverse reaction associated with a vaccine and/or severe allergic reaction (eg, anaphylaxis) to any component of the study intervention or any related vaccine.

- Current pregnancy resulting from in vitro fertilization. Participants known to have used clomiphene citrate and/or letrozole with or without intrauterine insemination were permitted.

- Current pregnancy complications or abnormalities at the time of consent that increase risk associated with the participation in and completion of the study, including but not limited to preeclampsia, eclampsia, or uncontrolled gestational hypertension; placental abnormality; polyhydramnios or oligohydramnios; significant bleeding or blood clotting disorder; and endocrine disorders, including untreated hyperthyroidism or untreated hypothyroidism. This also included disorders of glucose intolerance (eg, diabetes mellitus type 1 or 2) antedating pregnancy or occurring during pregnancy if uncontrolled at the time of consent.

- Any signs of premature labor with the current pregnancy or having ongoing intervention (medical/surgical) in the current pregnancy to prevent preterm birth.

- Previous pregnancy complications or abnormalities at the time of consent, based on the investigator's judgment, that increase the risk associated with the participation in and completion of the study, including but not limited to previous preterm delivery at ≤34 weeks' gestation; previous stillbirth or neonatal death; and previous infant with a known genetic disorder or significant congenital anomaly.

- Major illness of the maternal participant or conditions of the fetus that could substantially increase the risk associated with the maternal or infant participant's participation in, and completion of, the study or that could preclude the evaluation of the maternal participant's response (included positive serologic testing for regional endemic conditions assessed during routine maternal care, as per local standards of care and obstetric recommendations).

- Non‑HIV-associated congenital or acquired immunodeficiency disorder, or rheumatologic disorder or other illness requiring chronic treatment with known immunosuppressant medications.

Previous and concomitant therapy

- Participation in other studies involving investigational drug(s) within 28 days before consent and/or during study participation.

- Participation in any study involving maternal vaccination with a respiratory syncytial virus (RSV) vaccine or RSV monoclonal antibody (criterion applied to participants known to have been in receipt of the active study intervention arm in a previous study).

- Receipt of monoclonal antibodies within the year before enrollment or use of systemic corticosteroids for >14 days within 28 days before study enrollment. Permitted treatments included receipt of SARS-CoV-2 monoclonal antibodies; prednisone doses of <20 mg/day for ≤14 days; and inhaled/nebulized, intra-articular, intrabursal, or topical (skin or eyes) corticosteroids.

- Current anti-TB treatment or use at any time during the current pregnancy.

- Current alcohol abuse or illicit drug use. Marijuana use was not considered an exclusion criterion for the study when elicited in participant screening, although it could be considered illicit in some locales.

- Receipt of blood or plasma products or immune globulin from 60 days before study intervention administration, or planned receipt through delivery, apart from Rho(D) immune globulin (eg, RhoGAM), which could be given at any time.

Previous or concurrent clinical study experience

- Previous vaccination with any licensed or investigational RSV vaccine or planned receipt during study participation.

Other criterion

Participants who were breastfeeding at the time of enrollment.

Ethical study conduct

This study was conducted in accordance with the protocol and with the consensus ethical principles derived from international guidelines, including the Declaration of Helsinki and Council for International Organizations of Medical Sciences International Ethical Guidelines; applicable International Council for Harmonisation of Technical Requirements for Pharmaceuticals for Human Use Good Clinical Practice guidelines; and applicable laws and regulations, including applicable privacy laws.

Adverse events of special interest in maternal participants

Guillain-Barré syndrome included the adverse event (AE) preferred terms of acute motor axonal neuropathy, acute motor-sensory axonal neuropathy, ascending flaccid paralysis, Bickerstaff's encephalitis, chronic inflammatory demyelinating polyradiculoneuropathy, demyelinating polyneuropathy, Guillain-Barré syndrome, Miller Fisher syndrome, subacute inflammatory demyelinating polyneuropathy, and Zika virus associated Guillain-Barré syndrome.

Acute polyneuropathy included the AE preferred terms of acute polyneuropathy, autoimmune neuropathy, axonal and demyelinating polyneuropathy, axonal neuropathy, immune-mediated neuropathy, neuronal neuropathy, peripheral motor neuropathy, peripheral sensorimotor neuropathy, peripheral sensory neuropathy, and polyneuropathy.

Hypertensive disorders of pregnancy included the AE preferred terms of pre-eclampsia, eclampsia, gestational hypertension, Hemolysis, Elevated Liver enzymes and Low Platelets (HELLP) syndrome, and superimposed pre-eclampsia.

Preterm delivery (delivery at <37 0/7 weeks' gestation) included the AE preferred term of premature delivery.

Study sites

Botho Ke Bontle Health Services<br>Pretoria, Gauteng, South Africa	FAMCRU – Worcester<br>Worcester, Western Cape, South Africa
Gole Biomed Research Centre<br>Polokwane, Limpopo, South Africa	Gugulethu Green Clinic<br>Cape Town, Western Cape, South Africa
Josha Research<br>Bloemfontein, Free State, South Africa	MRC Unit on Child and Adolescent Health<br>Cape Town, Western Cape, South Africa
Qhakaza Mbokodo Research Clinic<br>Ladysmith, Kwazulu-Natal, South Africa	REIMED Reiger Park<br>Boksburg, Gauteng, South Africa
Setshaba Research Centre<br>Tshwane, Gauteng, South Africa	Synergy Biomed Research Institute<br>East London, Eastern Cape, South Africa
University of Witwatersrand (WITS) - Vaccines and Infectious Diseases Analytics (VIDA)<br>Johannesburg, Gauteng, South Africa	Wits RHI<br>Johannesburg, Gauteng, South Africa
Wits VIDA Nkanyezi Research Unit<br>Johannesburg, Gauteng, South Africa	Worthwhile Clinical Trials<br>Benoni, Gauteng, South Africa
