# Supplementary material for: Safety, Tolerability, and Immunogenicity of RSVpreF Vaccine in Pregnant Individuals Living with HIV
Source: Vaccines (Basel). 2025 Dec 1;13(12):1218. doi: 10.3390/vaccines13121218 (PMC12737651; doi:10.3390/vaccines13121218)
Supplement: Supplementary file 1 [file vaccines-13-01218-s001.zip › Table S1.pdf]

Table S1. Severity grading of local reactions, systemic events, and fever

	Mild	Moderate	Severe	Grade 4
**Local reaction**				
Redness	>2.0–5.0 cm	>5.0–10.0 cm	>10 cm	Necrosis or exfoliative dermatitis
Swelling	>2.0–5.0 cm	>5.0–10.0 cm	>10 cm	Necrosis
Injection site pain	Does not interfere with activity	Interferes with activity	Prevents daily activity	ED visit or hospitalization
**Systemic event**				
Fatigue	Does not interfere with activity	Some interference with activity	Prevents daily routine activity	ED visit or hospitalization
Headache	Does not interfere with activity	Some interference with activity	Prevents daily routine activity	ED visit or hospitalization
Vomiting	1–2 times in 24 hours	>2 times in 24 hours	Requires IV hydration	ED visit or hospitalization
Nausea	Does not interfere with activity	Some interference with activity	Prevents daily routine activity	ED visit or hospitalization
Diarrhea	2–3 loose stools in 24 hours	4–5 loose stools in 24 hours	6 or more loose stools in 24 hours	ED visit or hospitalization
Muscle pain	Does not interfere with activity	Some interference with activity	Prevents daily routine activity	ED visit or hospitalization
Joint pain	Does not interfere with activity	Some interference with activity	Prevents daily routine activity	ED visit or hospitalization
**Fever**				
Fever	38.0°C–38.4°C	>38.4°C –38.9°C	>38.9°C–40.0°C	>40.0°C

ED, emergency department; IV, intravenous.
