# Supplementary material for: Safety, Tolerability, and Immunogenicity of RSVpreF Vaccine in Pregnant Individuals Living with HIV
Source: Vaccines (Basel). 2025 Dec 1;13(12):1218. doi: 10.3390/vaccines13121218 (PMC12737651; doi:10.3390/vaccines13121218)
Supplement: Supplementary file 1 [file vaccines-13-01218-s001.zip › Table S2.pdf]

Table S2. Demographics for maternal participants living with HIV (current study) and non-HIV participants (MATISSE)

Maternal demographic characteristic	HIV Participant RSVpreF (N=144)	Non-HIV Participant RSVpreF (MATISSE) (N=415)
Age at vaccination, years		
Mean (SD)	31.2 (5.85)	27.1 (5.49)
Median (range)	31.0 (19- 44)	27.0 (17- 42)
Gestational age at vaccination, weeks		
Mean (SD)	29.8 (3.77)	29.8 (3.37)
Median (range)	30.30 (24.0, 36.0)	29.60 (24.0, 36.0)
Gestational age at vaccination, weeks		
≥24 weeks to <28 weeks	47 (32.6)	145 (34.9)
≥28 weeks to <32 weeks	43 (29.9)	139 (33.5)
≥32 weeks to ≤36 weeks	54 (37.5)	131 (31.6)
Vaccination to delivery interval, days		
Mean (SD)	64.78 (28.672)	66.89 (25.138)
Median (Range)	62.50 (2.0, 116.0)	66.00 (14.0, 121.0)

GA, gestational age.

Data are for the evaluable immunogenicity population.
