# Supplementary material for: Safety, Tolerability, and Immunogenicity of RSVpreF Vaccine in Pregnant Individuals Living with HIV
Source: Vaccines (Basel). 2025 Dec 1;13(12):1218. doi: 10.3390/vaccines13121218 (PMC12737651; doi:10.3390/vaccines13121218)
Supplement: Supplementary file 1 [file vaccines-13-01218-s001.zip › Table S3.pdf]

Table S3. HIV viral load and CD4 count for maternal participants at vaccination and at delivery

**Timepoint**<br>Parameter, N (%)	**RSVpreF (N=172)**	**Placebo (N=170)**
**Vaccination**		
HIV RNA lower than detectable limit	141 (82.0)	139 (81.8)
Not done	0	0
CD4 count >500 cells/mm^3	116 (67.4)	118 (69.4)
**Delivery***		
HIV RNA lower than detectable limit	137 (79.7)	135 (79.4)
HIV viral load of >1000 copies/mL	5 (2.9)	2 (1.2)
Not done	9 (5.2)	13 (7.7)
CD4 count >500 cells/mm^3	91 (52.9)	80 (47.1)

Data are for the safety population. *Data were not collected from all participants at delivery.
