# Supplementary material for: Safety, Tolerability, and Immunogenicity of RSVpreF Vaccine in Pregnant Individuals Living with HIV
Source: Vaccines (Basel). 2025 Dec 1;13(12):1218. doi: 10.3390/vaccines13121218 (PMC12737651; doi:10.3390/vaccines13121218)
Supplement: Supplementary file 1 [file vaccines-13-01218-s001.zip › Table S4.pdf]

Table S4. Serious adverse events in maternal recipients (reported from vaccination throughout the study) and for infant participants) reported through 1 month and 6 months after birth) by system organ class and preferred term*

System organ class<br>Preferred term	RSVpreF<br>(N=172)	Placebo<br>(N=170)
**Maternal participants**		
**Any event**	**60 (34.9)**	**57 (33.5)**
**Blood and lymphatic system disorders**	**1 (0.6)**	**0**
**Cardiac disorders**	**2 (1.2)**	**2 (1.2)**
**Gastrointestinal disorders**	**0**	**3 (1.8)**
**Hepatobiliary disorders**	**1 (0.6)**	**1 (0.6)**
**Infections and infestations**	**5 (2.9)**	**2 (1.2)**
**Injury, poisoning and procedural complications**	**1 (0.6)**	**3 (1.8)**
**Pregnancy, puerperium and perinatal conditions**	**54 (31.4)**	**51 (30.0)**
Breech presentation	3 (1.7)	1 (0.6)
Cephalo-pelvic disproportion	2 (1.2)	5 (2.9)
False labour	0	2 (1.2)
Fetal distress syndrome	25 (14.5)	22 (12.9)
Fetal growth restriction	1 (0.6)	2 (1.2)
Fetal macrosomia	0	2 (1.2)
Gestational hypertension	6 (3.5)	4 (2.4)
Obstructed labour	2 (1.2)	0
Postpartum haemorrhage	2 (1.2)	1 (0.6)
Pre-eclampsia	4 (2.3)	8 (4.7)
Premature delivery†	3 (1.7)	3 (1.8)
Preterm premature rupture of membranes	3 (1.7)	0
Prolonged labor	5 (2.0)	0
Prolonged rupture of membranes	2 (1.2)	1 (0.6)
Stillbirth	1 (0.6)	2 (1.2)
**Psychiatric disorders**	**1 (0.6)**	**0**
**Renal and urinary disorders**	**1 (0.6)**	**1 (0.6)**
**Reproductive system and breast disorders**	**2 (1.2)**	**2 (1.2)**
Uterine scar	2 (1.2)	2 (1.2)
**Respiratory, thoracic and mediastinal disorders**	**1 (0.6)**	**0**
**Social circumstances**	**0**	**1 (0.6)**
**Vascular disorders**	**0**	**1 (0.6)**
**Infant participants: birth to 1 month of age**		
**Any event**	**23 (13.5)**	**31 (18.7)**
**Blood and lymphatic system disorders**	**0**	**1 (0.6)**
**Cardiac disorders**	**1 (0.6)**	**0**
**Congenital, familial and genetic disorders**	**6 (3.5)**	**8 (4.8)**
Trisomy 21	0	2 (1.2)
**Gastrointestinal disorders**	**1 (0.6)**	**1 (0.6)**
**General disorders and administration site conditions**	**0**	**1 (0.6)**

**Infections and infestations**	**4 (2.3)**	**4 (2.4)**
Sepsis neonatal	2 (1.2)	1 (0.6)
**Injury, poisoning and procedural complications**	**1 (0.6)**	**0**
**Investigations**	**0**	**2 (1.2)**
**Metabolism and nutrition disorders**	**3 (1.8)**	**0**
**Musculoskeletal and connective tissue disorders**	**0**	**1 (0.6)**
**Nervous system disorders**	**0**	**3 (1.8)**
Encephalopathy neonatal	0	2 (1.2)
**Pregnancy, puerperium and perinatal conditions**	**9 (5.3)**	**9 (5.4)**
Jaundice neonatal	4 (2.3)	5 (3.0)
Low birth weight baby	2 (1.2)	5 (3.0)
Premature baby‡	3 (1.8)	5 (3.0)
**Renal and urinary disorders**	**1 (0.6)**	**0**
**Respiratory, thoracic and mediastinal disorders**	**6 (3.5)**	**13 (7.8)**
Meconium aspiration syndrome	0	2 (1.2)
Neonatal respiratory distress	2 (1.2)	2 (1.2)
Neonatal respiratory distress syndrome	3 (1.8)	3 (1.8)
Transient tachypnea of the newborn	0	4 (2.4)
**Infant participants: 1 month to 6 months of age**		
**Any event**	**9 (5.3)**	**11 (6.6)**
**Blood and lymphatic system disorders**	**0**	**2 (1.2)**
Anemia	0	2 (1.2)
**Endocrine disorders**	**1 (0.6)**	**0**
**Eye disorders**	**0**	**1 (0.6)**
**Gastrointestinal disorders**	**0**	**1 (0.6)**
**Infections and infestations**	**6 (3.5)**	**9 (5.4)**
Gastroenteritis	3 (1.8)	2 (1.2)
Pneumonia	2 (1.2)	1 (0.6)
**Investigations**	**1 (0.6)**	**0**
**Metabolism and nutrition disorders**	**2 (1.2)**	**1 (0.6)**
**Nervous system disorders**	**2 (1.2)**	**1 (0.6)**
**Respiratory, thoracic and mediastinal disorders**	**0**	**2 (1.2)**

Data are for the safety population.*Preferred terms are reported for serious adverse events occurring in >1 participant in either group. †Referred to as ‘preterm delivery’ in the text. ‡Referred to as ‘preterm birth’ in the text.
