# Supplementary material for: Safety, Tolerability, and Immunogenicity of RSVpreF Vaccine in Pregnant Individuals Living with HIV
Source: Vaccines (Basel). 2025 Dec 1;13(12):1218. doi: 10.3390/vaccines13121218 (PMC12737651; doi:10.3390/vaccines13121218)
Supplement: Supplementary file 1 [file vaccines-13-01218-s001.zip › Table S5.pdf]

Table S5. Characteristics of maternal participants with hypertension and hypertensive disorders of pregnancy

Characteristic	RSVpreF (N=12)	Placebo (N=15)	Total (N=27)
GA at birth, n (%)			
28–<34 weeks	0	1 (6.7)	1 (3.7)
34–<37 weeks	3 (25.0)	4 (26.7)	7 (25.9)
37–<42 weeks	9 (75.0)	9 (60.0)	18 (66.7)
≥42 weeks	0	1 (6.7)	1 (3.7)
Relative days of diagnosis from vaccination, n (%)			
≤7 days	2 (16.7)	0	2 (7.4)
>7–30 days	2 (16.7)	5 (33.3)	7 (25.9)
>30 days	8 (66.7)	10 (66.7)	18 (66.7)
Adverse event preferred terms			
Postpartum hypertension	2 (16.7)	0	2 (7.4)
Gestational hypertension	9 (75.0)	8 (53.3)	17 (63.0)
Pre-eclampsia	4 (33.3)	8 (53.3)	12 (44.4)
HELLP syndrome	0	0	0
Eclampsia	0	0	0
Serious adverse event	10 (83.3)	12 (80.0)	22 (81.5)

GA, gestational age; HELLP, Hemolysis, Elevated Liver enzymes and Low Platelets.
Data are for the safety population.
