# Supplementary material for: Safety, Tolerability, and Immunogenicity of RSVpreF Vaccine in Pregnant Individuals Living with HIV
Source: Vaccines (Basel). 2025 Dec 1;13(12):1218. doi: 10.3390/vaccines13121218 (PMC12737651; doi:10.3390/vaccines13121218)
Supplement: Supplementary file 1 [file vaccines-13-01218-s001.zip › Table S6.pdf]

Table S6. Characteristics of maternal participants with preterm and term delivery

	Preterm delivery			Term delivery			Total		
	**RSVpreF (N=15)**	**Placebo (N=13)**	**Total (N=28)**	**RSVpreF (N=156)**	**Placebo (N=153)**	**Total (N=309)**	**RSVpreF (N=171)**	**Placebo (N=166)**	**Total (N=337)**
Age at vaccination, years									
Mean (SD)	31.5 (6.85)	30.9 (5.36)	31.2 (6.10)	31.3 (5.70)	30.8 (6.01)	31.1 (5.85)	31.3 (5.78)	30.8 (5.95)	31.1 (5.86)
Median (range)	30 (22–42)	31 (20–38)	31 (20–42)	31.5 (19–44)	31 (16–43)	31 (16–44)	31 (19–44)	31 (16–43)	31 (16–44)
Relative time of delivery from vaccination, days									
≤7	2 (13.3)	0	2 (7.1)	0	0	0	2 (1.2)	0	2 (0.6)
>7–30	5 (33.3)	6 (46.2)	11 (39.3)	10 (6.4)	8 (5.2)	18 (5.8)	15 (8.8)	14 (8.4)	29 (8.6)
>30	8 (53.3)	7 (53.8)	15 (53.6)	146 (93.6)	145 (94.8)	291 (94.2)	154 (90.1)	152 (91.6)	306 (90.8)
GA at delivery, weeks									
24–<28	0	1 (7.7)	1 (3.6)	0	0	0	0	1 (0.6)	1 (0.3)
28–<34	1 (6.7)	2 (15.4)	3 (10.7)	0	0	0	1 (0.6)	2 (1.2)	3 (0.9)
34–<37	14 (93.3)	10 (76.9)	24 (85.7)	0	0	0	14 (8.2)	10 (6.0)	24 (7.1)
37–<42	0	0	0	155 (99.4)	147 (96.1)	302 (97.7)	155 (90.6)	147 (88.6)	302 (89.6)
≥42	0	0	0	1 (0.6)	6 (3.9)	7 (2.3)	1 (0.6)	6 (3.6)	7 (2.1)
Maternal obstetrical history by number of previous pregnancies, n (%)									
0	2 (13.3)	0	2 (7.1)	24 (15.4)	28 (18.3)	52 (16.8)	26 (15.2)	28 (16.9)	54 (16.0)
1–3	10 (66.7)	12 (92.3)	22 (78.6)	120 (76.9)	112 (73.2)	232 (75.1)	130 (76.0)	124 (74.7)	254 (75.4)
≥4	3 (20.0)	1 (7.7)	4 (14.3)	12 (7.7)	13 (8.5)	25 (8.1)	15 (8.8)	14 (8.4)	29 (8.6)
Maternal obstetrical history by number of previous live births, n (%)									
0	3 (20.0)	1 (7.7)	4 (14.3)	32 (20.5)	32 (20.9)	64 (20.7)	35 (20.5)	33 (19.9)	68 (20.2)
1–3	11 (73.3)	11 (84.6)	22 (78.6)	121 (77.6)	115 (75.2)	236 (76.4)	132 (77.2)	126 (75.9)	258 (76.6)
≥4	1 (6.7)	1 (7.7)	2 (7.1)	3 (1.9)	6 (3.9)	9 (2.9)	4 (2.3)	7 (4.2)	11 (3.3)
Maternal nonstudy vaccination reported before delivery, n (%)									

0	6 (40.0)	3 (23.1)	9 (32.1)	45 (28.8)	42 (27.5)	87 (28.2)	51 (29.8)	45 (27.1)	96 (28.5)
≥1	9 (60.0)	10 (76.9)	19 (67.9)	111 (71.2)	111 (72.5)	222 (71.8)	120 (70.2)	121 (72.9)	241 (71.5)
Estimated due date timing, n (%)									
First trimester	3 (20.0)	1 (7.7)	4 (14.3)	38 (24.4)	36 (23.5)	74 (23.9)	41 (24.0)	37 (22.3)	78 (23.1)
Second trimester	12 (80.0)	12 (92.3)	24 (85.7)	118 (75.6)	117 (76.5)	235 (76.1)	130 (76.0)	129 (77.7)	259 (76.9)
Birthweight, n (%)									
Extremely low (≤1000 g)	0	1 (7.7)	1 (3.6)	0	0	0	0	1 (0.6)	1 (0.3)
Very low (1001–1500 g)	1 (6.7)	1 (7.7)	2 (7.1)	0	0	0	1 (0.6)	1 (0.6)	2 (0.6)
Low (1501–2500 g)	9 (60.0)	4 (30.8)	13 (46.4)	13 (8.3)	15 (9.8)	28 (9.1)	22 (12.9)	19 (11.4)	41 (12.2)
Normal (>2500 g)	5 (33.3)	7 (53.8)	12 (42.9)	141 (90.4)	135 (88.2)	276 (89.3)	146(85.4)	142 (85.5)	288 (85.5)
Causality, n (%)									
Related to study vaccination	0	1 (7.7)	1 (3.6)	0	0	0	0	1 (0.6)	1 (0.3)
Not related to study vaccination	15 (100.0)	12 (92.3)	27 (96.4)	0	0	0	15 (8.8)	12 (7.2)	27 (8.0)
Pathway to preterm delivery, n (%)									
Spontaneous	5 (33.3)	9 (69.2)	14 (50.0)	0	0	0	5 (2.9)	9 (5.4)	14 (4.2)
Preterm premature rupture of membranes	3 (20.0)	0	3 (10.7)	0	0	0	3 (1.8)	0	3 (0.9)
Provider initiated	6 (40.0)	3 (23.1)	9 (32.1)	0	0	0	6 (3.5)	3 (1.8)	9 (2.7)
Unknown	1 (6.7)	0	1 (3.6)	0	0	0	1 (0.6)	0	1 (0.3)

GA, gestational age.
