# Supplementary material for: Safety, Tolerability, and Immunogenicity of RSVpreF Vaccine in Pregnant Individuals Living with HIV
Source: Vaccines (Basel). 2025 Dec 1;13(12):1218. doi: 10.3390/vaccines13121218 (PMC12737651; doi:10.3390/vaccines13121218)
Supplement: Supplementary file 1 [file vaccines-13-01218-s001.zip › Table S7.pdf]

Table S7. RSV neutralizing titer GMRs before vaccination and at delivery (maternal participants) and birth (infant participants)

RSV subgroup	Timepoint	RSVpreF vs placebo GMR (95% CI)
Maternal participants (RSVpreF, N=144; placebo, N=139)		
A	Before vaccination	0.99 (0.83, 1.19)
	At delivery	7.93 (6.44, 9.77)
B	Before vaccination	1.08 (0.88, 1.33)
	At delivery	7.15 (5.78, 8.84)
A/B	Before vaccination	1.04 (0.86, 1.24)
	At delivery	7.53 (6.2, 9.15)
Infant participants (RSVpreF, N=154; placebo, N=143–145)		
A	At birth	7.77 (6.36, 9.50)
B	At birth	6.78 (5.41, 8.50)
A/B	At birth	7.21 (5.91, 8.80)

GMR, geometric mean ratio.

GMRs were calculated as the group mean difference of logarithmically transformed antibody levels and back transformed to the original units. CIs were back transformations of CIs based on the Student *t* distribution for the mean difference of logarithm of the titers. For each individual, combined RSV-A/RSV-B was calculated as the geometric mean of the titer of RSV-A and RSV-B at the specified timepoint.
