# Supplementary material for: Safety, Tolerability, and Immunogenicity of RSVpreF Vaccine in Pregnant Individuals Living with HIV
Source: Vaccines (Basel). 2025 Dec 1;13(12):1218. doi: 10.3390/vaccines13121218 (PMC12737651; doi:10.3390/vaccines13121218)
Supplement: Supplementary file 1 [file vaccines-13-01218-s001.zip › Table S8.pdf]

Table S8. Infant RSV neutralizing GMTs and GMRs at birth by maternal gestational age at vaccination

Gestational age at vaccination	RSV subgoup	Study intervention group						Comparison
		RSVpreF			Placebo			RSVpreF vs placebo
		n	GMT	(95% CI)	n	GMT	(95% CI)	GMR (95% CI)
24−<28 weeks	A	52	9939	(7802.7, 12,660.6)	50	1715	(1403.4, 2096.5)	5.79 (4.24, 7.91)
	B	52	11,343	(8566.5, 15,020.2)	49	2274	(1816.3, 2846.7)	4.99 (3.49, 7.13)
	A/B	52	10,618	(8289.4, 13,600.8)	49	1977	(1613.3, 2422.0)	5.37 (3.91, 7.39)
28−<32 weeks	A	48	13,571	(9920.4, 18,565.1)	51	1354	(1062.3, 1725.1)	10.02 (6.80, 14.78)
	B	48	14,301	(10,727.9, 19,064.4)	51	1658	(1253.8, 2191.6)	8.63 (5.81, 12.82)
	A/B	48	13,931	(10,441.3, 18,587.8)	51	1498	(1173.4, 1912.3)	9.30 (6.41, 13.48)
32−36 weeks	A	54	14,627	(11,740.1, 18,223.9)	44	1846	(1407.3, 2420.2)	7.93 (5.64, 11.14)
	B	54	16,292	(12,474.8, 21,278.4)	45	2300	(1625.9, 3253.9)	7.08 (4.63, 10.84)
	A/B	54	15,437	(12,265.4, 19,429.5)	43	2114	(1623.7, 2751.3)	7.30 (5.18, 10.31)

GMR, geometric mean ratio; GMT, geometric mean titer; LLOQ, lower limit of quantitation; RSV, respiratory syncytial virus.

This subgroup analysis was conducted in the infant evaluable immunogenicity population.

The LLOQ values were 242 for RSV-A and 99 for RSV-B neutralizing titers. Assay results below the LLOQ were set to 0.5 × LLOQ.

GMTs and 2-sided 95% CIs were calculated by exponentiating the mean logarithm of the titers and the corresponding CIs (based on the Student *t* distribution).

GMRs were calculated as the group mean difference of logarithmically transformed antibody levels and back transformed to the original units. The associated 95% CIs were back transformations of CIs based on the Student *t* distribution for the mean difference of logarithm of the titers. For each individual, combined RSV-A/RSV-B was calculated as the geometric mean of the titer of RSV-A and RSV-B at the specified timepoint.
