# Supplementary material for: Safety, Tolerability, and Immunogenicity of RSVpreF Vaccine in Pregnant Individuals Living with HIV
Source: Vaccines (Basel). 2025 Dec 1;13(12):1218. doi: 10.3390/vaccines13121218 (PMC12737651; doi:10.3390/vaccines13121218)
Supplement: Supplementary file 1 [file vaccines-13-01218-s001.zip › Table S9.pdf]

Table S9. Maternal unadjusted RSV neutralizing GMTs and GMRs by gestational age at vaccination

RSV subgoup	Gestational age at vaccination (weeks)	Time point	Study intervention group						Comparison
			HIV participants RSVpreF			Non-HIV participants RSVpreF			HIV vs non-HIV
			n	GMT	(95% CI)	n	GMT	(95% CI)	GMR (95% CI)
RSV-A	24 to <28	Before vaccination	47	1474	(1174.1, 1851.5)	144	1353	(1214.5, 1507.2)	1.09 (0.97, 1.37)
		At Delivery	47	9869	(7568.3, 12,869.7)	145	12,063	(10,669.0, 13,638.9)	0.82 (0.63, 1.06)
	28 to <32	Before vaccination	43	1608	(1292.7, 1999.6)	139	1405	(1252.7, 1575.3)	1.14 (0.90, 1.45)
		At Delivery	43	11,977	(9119.5, 15,730.6)	139	12,960	(11,460.6, 14,655.9)	0.92 (0.71, 1.21)
	32 to ≤36	Before vaccination	54	1856	(1448.9, 2377.7)	131	1473	(1313.5, 1652.6)	1.26 (0.99, 1.60)
		At Delivery	54	18,342	(14,523.4, 23,164.8)	131	19,181	(16,825.0, 21,866.1)	0.96 (0.74, 1.23)
RSV-B	24 to <28	Before vaccination	47	2043	(1587.2, 2628.8)	144	1853	(1665.7, 2061.1)	1.10 (0.87, 1.39)
		At Delivery	47	14,323	(11,086.1, 18,505.7)	145	15,459	(13,523.5, 17,672.5)	0.93 (0.70, 1.22)
	28 to <32	Before vaccination	43	1899	(1481.7, 2433.2)	139	2045	(1797.7, 2325.8)	0.93 (0.71, 1.21)
		At Delivery	43	12,909	(9722.0, 17,140.9)	139	18,938	(16,316.3, 21,980.5)	0.68 (0.50, 0.93)
	32 to ≤36	Before vaccination	54	2775	(2148.0, 3585.6)	131	1933	(1692.0, 2207.3)	1.44 (1.10, 1.87)
		At Delivery	54	22,413	(16,962.6, 29,614.8)	131	26,387	(22,749.0, 30,607.7)	0.85 (0.64, 1.13)
RSV-A/B	24 to <28	Before vaccination	47	1735	(1392.1, 2163.4)	144	1583	(1438.6, 1742.6)	1.10 (0.89, 1.35)
		At Delivery	47	11,889	(9438.7, 14,976.6)	145	13,656	(12,139.5, 15,361.9)	0.87 (0.68, 1.11)
	28 to <32	Before vaccination	43	1747	(1399.8, 2180.9)	139	1695	(1514.1, 1897.1)	1.03 (0.81, 1.30)
		At Delivery	43	12,434	(9524.7, 16,233.1)	139	15,666	(13,850.2, 17,720.9)	0.79 (0.61, 1.03)
	32 to ≤36	Before vaccination	54	2270	(1780.6, 2892.9)	131	1687	(1504.9, 1892.0)	1.35 (1.06, 1.70)
		At Delivery	54	20,276	(15,963.9, 25,751.9)	131	22,497	(19,822.8, 25,532.7)	0.90 (0.70, 1.15)

GMR, geometric mean ratio; GMT, geometric mean titer; RSV, respiratory syncytial virus.

Data are for the evaluable immunogenicity population.
