# Supplementary material for: Safety, Tolerability, and Immunogenicity of RSVpreF Vaccine in Pregnant Individuals Living with HIV
Source: Vaccines (Basel). 2025 Dec 1;13(12):1218. doi: 10.3390/vaccines13121218 (PMC12737651; doi:10.3390/vaccines13121218)
Supplement: Supplementary file 1 [file vaccines-13-01218-s001.zip › Table S10.pdf]

Table S10. Infant unadjusted RSV neutralizing GMTs and GMRs at birth by maternal gestational age at vaccination

RSV subgoup	Gestational age at vaccination (weeks)	Study intervention group						Comparison
		HIV participants RSVpreF			Non-HIV participants RSVpreF			HIV vs non-HIV
		n	GMT	(95% CI)	n	GMT	(95% CI)	GMR (95% CI)
RSV-A	24 to <28	52	9939	(7802.7, 12,660.6)	152	14,258	(12,461.8, 16,313.2)	0.70 (0.53, 0.91)
	28 to <32	48	13,571	(9920.4, 18,565.1)	150	14,720	(12,858.7, 16,850.2)	0.92 (0.69, 1.24)
	32 to ≤36	54	14,627	(11,740.1, 18,223.9)	134	16,637	(14,444.0, 19,163.9)	0.88 (0.68, 1.14)
RSV-B	24 to <28	52	11,343	(8566.5, 15,020.2)	152	18,481	(16,000.1, 21,347.2)	0.61 (0.46, 0.82)
	28 to <32	48	14,301	(10,727.9, 19,064.4)	150	19,518	(16,661.9, 22,862.6)	0.73 (0.53, 1.01)
	32 to ≤36	54	16,292	(12,474.8, 21,278.4)	134	22,104	(18,988.2, 25,732.2)	0.74 (0.55, 0.99)
RSV-A/B	24 to <28	52	10,618	(8289.4, 13,600.8)	152	16,233	(14,272.2, 18,463.0)	0.65 (0.50, 0.85)
	28 to <32	48	13,931	(10,441.3, 18,587.8)	150	16,950	(14,791.1, 19,423.5)	0.82 (0.62, 1.10)
	32 to ≤36	54	15,437	(12,265.4, 19,429.5)	134	19,177	(16,730.2, 21,981.9)	0.80 (0.62, 1.04)

GMR, geometric mean ratio; GMT, geometric mean titer; RSV, respiratory syncytial virus.

Data are for the evaluable immunogenicity population.
