# Supplementary material for: Safety, Tolerability, and Immunogenicity of RSVpreF Vaccine in Pregnant Individuals Living with HIV
Source: Vaccines (Basel). 2025 Dec 1;13(12):1218. doi: 10.3390/vaccines13121218 (PMC12737651; doi:10.3390/vaccines13121218)
Supplement: Supplementary file 1 [file vaccines-13-01218-s001.zip › Table S11.pdf]

Table S11. Maternal unadjusted RSV neutralizing GMTs and GMRs by maternal CD4 levels

RSV subgoup	Time point	CD4 >500 cells/mm³ (Visit 1 or Visit 3)	Study intervention group						Comparison
			RSVpreF			Placebo			RSVpreF vs placebo
			n	GMT	(95% CI)	n	GMT	(95% CI)	GMR (95% CI)
RSV-A	Before vaccination	Yes	110	1632	(1411.1, 1888.2)	103	1588	(1389.3, 1815.6)	1.03 (0.84, 1.25)
		No	34	1706	(1230.3, 2366.0)	36	1895	(1372.9, 2614.7)	0.90 (0.57, 1.41)
	At Delivery	Yes	110	13,886	(11,780.7, 16,368.3)	103	1594	(1353.7, 1878.0)	8.71 (6.91, 10.97)
		No	34	11,176	(7771.8, 16,072.8)	36	1875	(1372.9, 2561.4)	5.96 (3.73, 9.52)
RSV-B	Before vaccination	Yes	110	2222	(1894.1, 2605.6)	103	1950	(1674.9, 2271.3)	1.14 (0.91, 1.42)
		No	34	2310	(1617.9, 3297.1)	36	2476	(1733.3, 3536.9)	0.93 (0.57, 1.53)
	At Delivery	Yes	110	17,391	(14,540.6, 20,799.5)	103	2255	(1936.4, 2626.0)	7.71 (6.10, 9.76)
		No	34	13,650	(9520.8, 19,570.3)	36	2422	(1729.2, 3392.2)	5.64 (3.47, 9.14)
RSV-A/B	Before vaccination	Yes	110	1904	(1650.8, 2196.7)	103	1760	(1543.1, 2007.4)	1.08 (0.89, 1.31)
		No	34	1985	(1425.9, 2763.4)	36	2166	(1585.9, 2958.0)	0.92 (0.59, 1.43)
	At Delivery	Yes	110	15,540	(13,258.3, 18,214.4)	103	1896	(1637.9, 2195.3)	8.20 (6.61, 10.17)
		No	34	12,352	(8798.8, 17,338.8)	36	2131	(1581.4, 2872.0)	5.80 (3.72, 9.02)

GMR, geometric mean ratio; GMT, geometric mean titer; RSV, respiratory syncytial virus.

Data are for the evaluable immunogenicity population.
