# Supplementary material for: Safety, Tolerability, and Immunogenicity of RSVpreF Vaccine in Pregnant Individuals Living with HIV
Source: Vaccines (Basel). 2025 Dec 1;13(12):1218. doi: 10.3390/vaccines13121218 (PMC12737651; doi:10.3390/vaccines13121218)
Supplement: Supplementary file 1 [file vaccines-13-01218-s001.zip › Table S12.pdf]

Table S12. Infant unadjusted RSV neutralizing GMTs and GMRs at birth by maternal CD4 levels

RSV subgoup	CD4 >500 cells/mm^3 (Visit 1 or Visit 3)	Study intervention group						Comparison
		HIV participants RSVpreF			Non-HIV participants RSVpreF			HIV vs non-HIV
		n	GMT	(95% CI)	n	GMT	(95% CI)	GMR (95% CI)
RSV-A	Yes	118	13,529	(11,577.0, 15,810.4)	111	1549	(1335.5, 1797.2)	8.73 (7.05, 10.82)
	No	36	9782	(6703.3, 14,275.9)	34	1843	(1328.9, 2556.9)	5.31 (3.24, 8.69)
RSV-B	Yes	118	14,839	(12,534.3, 17,568.1)	110	2039	(1725.1, 2409.8)	7.28 (5.74, 9.22)
	No	36	11,024	(7373.6, 16,482.8)	35	2051	(1322.6, 3181.1)	5.37 (3.00, 9.64)
RSV-A/B	Yes	118	14,169	(12,143.6, 16,532.4)	110	1785	(1536.5, 2074.5)	7.94 (6.40, 9.84)
	No	36	10,385	(7174.3, 15,032.2)	33	1973	(1427.0, 2728.2)	5.26 (3.24, 8.56)

GA, gestational age; GMR, geometric mean ratio; GMT, geometric mean titer; RSV, respiratory syncytial virus.

Data are for the evaluable immunogenicity population.
