# Supplementary figures and images for: Safety, Tolerability, and Immunogenicity of RSVpreF Vaccine in Pregnant Individuals Living with HIV
Source: Vaccines (Basel). 2025 Dec 1;13(12):1218. doi: 10.3390/vaccines13121218 (PMC12737651; doi:10.3390/vaccines13121218)

**Figure S1. Disposition of maternal participants**

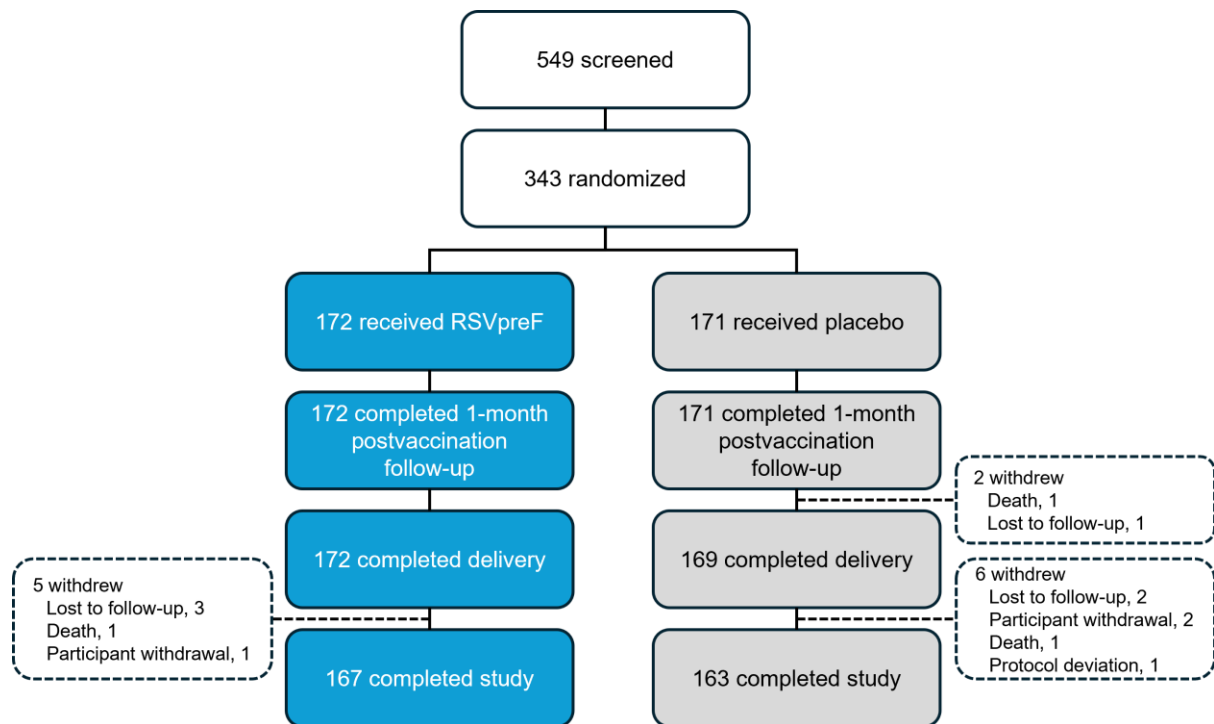

Supplement: Supplementary file 1 [file vaccines-13-01218-s001.zip › Figure S1.pdf]

**Figure S2. Disposition of infant participants**

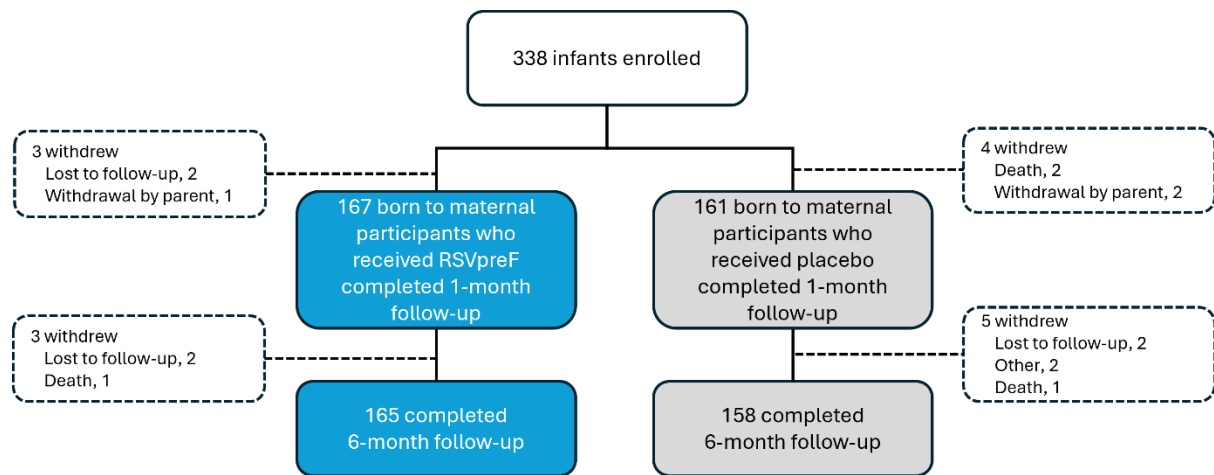

Supplement: Supplementary file 1 [file vaccines-13-01218-s001.zip › Figure S2.pdf]
